# H_2_O-rich rutile as an indicator for modern-style cold subduction

**DOI:** 10.1007/s00410-024-02107-2

**Published:** 2024-03-10

**Authors:** Mona Lueder, Renée Tamblyn, Daniela Rubatto, Jörg Hermann

**Affiliations:** 1 Institute of Geological Sciences, University of Bern, Baltzerstrasse 1+3, 3012 Bern, Switzerland; 2 Institute of Earth Sciences, University of Lausanne, Géopolis, Quartier Mouline, 1015 Lausanne, Switzerland

**Keywords:** Hydrogen in rutile, FTIR, Coldsubduction, Geobarometry

## Abstract

The trace-element and isotope geochemistry of rutile are robust tools to determine metamorphic temperatures, age, and host-/source lithologies. The use of rutile as single grain indicator for pressure, temperature, time and composition (P–T–t–X) of the host rock, which is vital in the use of detrital rutile to trace plate-tectonic regimes throughout Earth’s history, requires the identification of a pressure dependent trace element in rutile. We investigate the pressure dependence of hydrogen in rutile using polarized in-situ Fourier Transform Infrared (FTIR) spectroscopy. H_2_O contents in rutile vary between < 10–2500 μg/g H_2_O with higher contents in samples with higher peak metamorphic pressures, making H_2_O-in-rutile a viable pressure indicator. The highest H_2_O contents at ~ 450–2000 μg/g are observed in mafic low temperature eclogite-facies rutile related to modern-style cold subduction conditions. Hydrogen zoning in FTIR maps indicates that H^+^ is retained at temperatures below 600–700 °C. Ratios of H_2_O/Zr, using H_2_O as pressure indicator and Zr as temperature proxy, are a proxy for thermal gradients of metamorphic rutile (i.e. P/T). Low temperature eclogite samples are also characterized by high Fe contents and therefore Fe/Zr-ratios might be used as a first order approximation for H_2_O/Zr-ratios to identify mafic low temperature eclogite facies rutile. Based on common discrimination diagrams, Nb, W, and Sn can be used to distinguish different host/source rock lithologies of rutile. Combining both H_2_O/Zr-ratios and Nb, W, and Sn contents can thus identify modern-style cold subduction signatures in rutile. The developed systematics can consequently be used to trace cold-subduction features in the (pre-Proterozoic) detrital record.

**Supplementary Information:**

The online version contains supplementary material available at 10.1007/s00410-024-02107-2.

## Introduction

The evolution of plate tectonics on Earth and the onset of modern-style cold subduction (i.e. following subduction zone geotherms similar to the models for Honshu and Nicaragua of Syracuse et al. (2010), with gradients of ~ 150–350 °C/GPa) is a highly controversial and widely discussed topic in Earth sciences (e.g., Cawood et al. 2018; Holder et al. 2019; Brown et al. 2020). Modern-style subduction is characterized by conditions that produce low temperature (LT)–high pressure (HP) and ultra-high pressure (UHP) metamorphism, expressed as blueschist and eclogite facies rocks, and the formation of UHP minerals such as coesite and diamond (e.g. Stern 2018). Paired metamorphic belts, where a LT–HP subduction-related terrane is juxtaposed with a back-arc or orogenic hinterland, are also a representation of modern-style subduction (Miyashiro 1961). Thus, the occurrence of these LT–HP rock types in the geological record are considered diagnostic of the plate tectonic regime present on Earth. Some of the oldest subduction-related eclogites are Palaeoproterozoic in age (ca. 2.0–1.8 Ga; e.g., Brown and Johnson 2018), however generally formed along warmer thermal gradients than are present today, due to a warmer mantle temperature (e.g., Stern 2018), or are often retrogressed to granulite- or amphibolite facies conditions. Examples of preserved subduction-related eclogite-facies rock can be found in the the Trans-Hudson Orogen, North America (Weller and St-Onge 2017), the Snowbird Tectonic Zone, Canada (Baldwin et al. 2004), Nagssugtoqidian Orogen, Greenland (Glassley et al. 2014), Eburian-Transamazonian Orogen of southern Cameroon (Loose and Schenk 2018), the Congo Craton (François et al. 2018), the Usagaran and Ubendian Orogens, Tanzania (Möller et al. 1995; Collins et al. 2004; Boniface et al. 2012; Tamblyn et al. 2021), and the Belomoria Province, Russia (Herwartz et al. 2012; Liu et al. 2017; Xu et al. 2018; Yu et al. 2019). Metamorphic rocks recording cold modern-style subduction thermal gradients (similar to e.g. Honshu and Nicaragua of Syracuse et al. 2010) first occur at ca. 600 Ma and become more frequent in the late Neoproterozoic and Phanerozoic (e.g., Stern 2018; Brown and Johnson 2018, 2019; Brown et al. 2020). The oldest UHP terranes are even younger, with the oldest coesite-bearing eclogites known at ca. 620 Ma (e.g., Caby 1994; Ganade et al. 2023). However, the in-situ geological record is incomplete, particularly when going further back in time, due to erosion, overprinting, and crustal recycling. Thus, older modern-style cold subduction-related rocks might be missing from the rock record. Whether the absence of preserved cold-subduction-related rocks before the late Neoproterozoic is evidence of the onset of modern-style subduction around 600 Ma or a preservation bias is worthy of investigation. One way to do so is by probing the sedimentary record for accessory phases which potentially derive from eroded pre-Neoproterozoic LT–HP or UHP metamorphic rocks. This would require the determination of formation pressure and temperature, age, and composition of the source rock (P–T–t–X) on single detrital grains. However, a suitable phase for detrital single-grain P–T–t–X analyses has yet to be established.

Rutile is a prime candidate for the investigation of subduction processes through time from the sedimentary record. It is a common accessory metamorphic mineral in high-grade metamorphic rocks (HP metapelites, granulites, pressure-dominated amphibolites, blueschists, eclogites) and is very stable during chemical and mechanical weathering. Thus, rutile is a ubiquitous heavy mineral in sedimentary rocks. Additionally, the simple chemical composition of rutile (TiO_2_) makes it resistant to reaction processes even during burial and diagenesis, allowing it to survive multiple sedimentary cycles (e.g., Pereira and Storey 2023). Rutile can serve as petrogenetic indicator due to its ability to incorporate a variety of trace elements, partially dependant on the host rock or formation conditions, as such that discrimination diagrams can be used to infer the host rock. The incorporation of Zr into rutile is temperature dependent (e.g. Zack et al. 2004a; Tomkins et al. 2007; Kohn 2020) and Zr-in-rutile thermometry is frequently used in metamorphic and detrital studies. Rutile in high-grade metamorphic rocks can contain significant amounts of U (10–100 s μg/g), sufficient for U–Pb geochronology, which can be used in sedimentary provenance analyses (e.g., Pereira and Storey 2023). Niobium and Cr contents allow the differentiation of rutile from mafic and felsic lithologies (Zack et al. 2004b; Meinhold et al. 2008), whereas W, Sn, Sc, and Sb might be useful to identify rutile from felsic magmatic rocks and hydrothermal- or ore related mineralisation (e.g., Clark and Williams-Jones 2004; Meinhold 2010; Agangi et al. 2019; Pereira et al. 2021). However, formation pressures of metamorphic rutile cannot yet be determined from rutile chemistry. Thus, it is vital to firstly refine discrimination diagrams to reliably exclude non-metamorphic rutile from the detrital record, and secondly to develop a pressure indicator based on rutile trace-element geochemistry in order to use rutile as a tool to investigate subduction conditions in the sedimentary record.

One potential pressure indicator is H^+^. Among the nominally anhydrous minerals (NAMs), rutile is one of the most H_2_O-rich and can incorporate up to several 1000 μg/g H_2_O during subduction metamorphism (e.g., Soffer 1961; Rossman and Smyth 1990; Hammer and Beran 1991; Vlassopoulos et al. 1993; Zhang et al. 2001). Hydrogen is incorporated into rutile by coupled substitutions with di- and trivalent cations (e.g. Johnson et al. 1968; Vlassopoulos et al. 1993; Bromiley and Hilairet 2005). Natural rutile from different lithologies and P–T-conditions has been shown to incorporate H^+^ linked to six main di- and trivalent impurities, most commonly Ti^3+^ and Fe^3+^, to a lesser degree Al^3+^ and Mg^2+^, as well as Fe^2+^ and Cr^2+^ in reduced conditions (Lueder et al. 2023). However, the dominant H^+^-defects in rutile from different lithologies have not been evaluated. Water fugacity (${\text{f}}_{{{\text{H}}_{2} {\text{O}}}}$) might be an important variable which controls H^+^ incorperation into rutile. As ${\text{f}}_{{{\text{H}}_{2} {\text{O}}}}$ is pressure dependent, it may translate to pressure-dependent H_2_O contents in rutile. To investigate this, the use of polarised Fourier Transform Infrared Spectroscopy (FTIR) is a simple and precise tool to quantify H_2_O contents in randomly oriented rutile grains and can be applied to both metamorphic samples in-situ and single detrital grains (Lueder et al. 2023).

In this contribution, we evaluate the potential of H_2_O and trace elements in rutile as indicator for modern-style cold subduction conditions. We present results from FTIR spectroscopy and laser ablation inductively coupled plasma mass spectrometry (LA–ICP–MS) of rutile in metamorphic rocks from a variety of P–T conditions and lithologies including hydrothermal and pegmatitic rutile. We assess the importance of different defect types and the variability of H_2_O contents in rutile, depending on host rock and metamorphic conditions. FTIR mapping is presented to evaluate H^+^ retention in rutile from LT and HT lithologies. We also discuss the applicability of Nb, Cr, W, and Sn contents to identify rutile source lithologies. Furthermore, we evaluate the pressure dependence of H_2_O in rutile and its potential as a geochemical tool to identify cold thermal gradient metamorphism.

## Materials and methods

### Samples

**Fig. 1:**
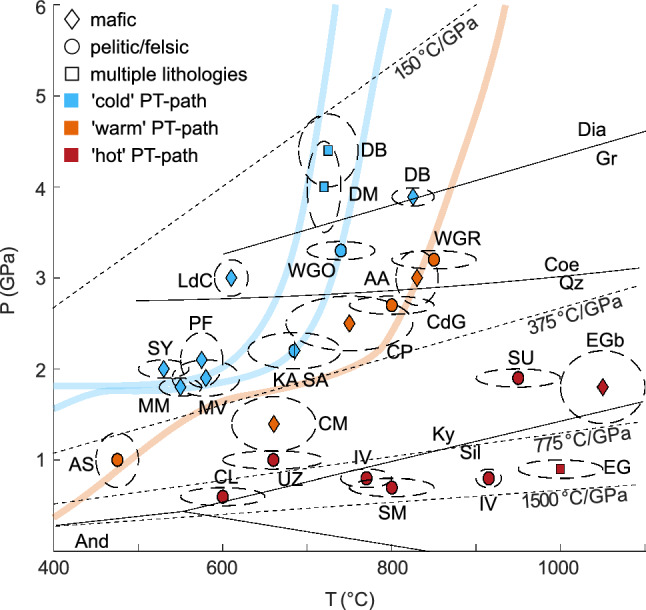
Peak P–T conditions of analysed samples. Diamonds indicate sample localities with mafic lithologies, circles indicate localities with felsic lithologies, and squares indicate sample localities with both mafic and felsic lithologies. Blue symbols indicate samples from cold subduction geotherms, similar to the D80 models for Honshu and Nicaragua (Syracuse et al. 2010), indicated as light blue lines. Orange symbols indicate sample localities related to warm geothermal gradients, similar to the D80 model for Cascadia (Syracuse et al. 2010), indicated as light orange line. Red symbols indicate localities related to hot geothermal gradients with thermal gradients of ~ 500–1500 °C/GPa. See Sect. 2 for literature references. *AA* Alpe Arami, *AS* Alpe Senevedo Superiore, *CM* Val Cama, *CL* Campolungo, *CP* Alpe Capoli, *CdG* Cima di Gagnone, *DB* Dabie Shan, *DM* Dora-Maira, *EG* Eastern Ghats, *EGb* Erzgebirge, *IV* Ivrea, *KA* Koralpe, *LdC* Lago di Cignana, *MM* Monte Mucrone, *PF* Pfulwe, *SA* Saualpe, *SM* Serre Massif, *SU* Sudetes, *SY* Syros, *UZ* Ulten Zone, *WGR* Western Gneiss Region, *WGO* West Gondwana Orogen. P–T conditions from literature, with reported uncertainties as elipses, for references see Table 1

We studied a total of 72 samples from 33 localities (Table 1, Fig. 1). The samples were chosen to represent a large subset of the rutile stability field in pressure, temperature and lithology. Detailed sample descriptions are given in Online Resource 1.

**Table 1:** List of metamorphic samples with sample localities, P–T, mineral assemblages and lithology

Locality	Sample	Mineral Assemblage	Lithology	P–T
Western Alps				
Dora-Maira	DM1c	Qz, Grt, Ky, Tlc, Rt, ± Ap, ± Zrc, ± Mca	Whiteschist	4.0 ± 0.5 GPa, 720 ± 20 °C ^(10, 13)^
	DM2a	Qz, Tlc, Grt, Ky, Rt, ± Ap, ± Zrc	Vein within Whiteschist	
	DM5	Mca, Grt, Qz, Ky, Rt, Amp ± Ap, ± Zrc	Metapelite	
	DM9	Grt, Omp, Qz, Rt, ± Aln, ± Ap, ± Zrc	Eclogite + Metapelite	
	DM10	Qz, Grt, Mca, Ky, Tlc, Rt, ± Ap, ± Zrc	Metapelite	
	DM30	Grt, Omp, Qz, Rt, ± Aln, ± Ap, ± Zrc	Eclogite	
Lago di Cignana	C31	Grt, Omp, Gln, Mca, Ca-Amp, Qz, Rt, ± Zrc	Eclogite	3.0 ± 0.2 GPa, 610 ± 20 °C ^(11, 22)^
Pfulwe	PF18-14	Grt, Gln, Pg, Omp, Rt, Cld, ± Ap, ± Zrc, ± Amp, ± Zrc	Fe-Ti-metagabbro	2.1 ± 0.3 GPa, 575 ± 25 °C ^(2)^
	PF18-25c	Grt, Omp, Ep, Pg, Mca, Rt, Qz, ± Chl, ± Gln, ± Zrc	Meta-pillowbasalt	
	PF18-26b	Grt, Gln, Omp, Pg, Qz, Rt, ± Ep, ± Zrc	Metagabbro	
	PF18TB01	Grt, Omp, Ep, Pg, Mca, Rt, Qz, ± Chl, ± Gln, ± Zrc	Meta-pillowbasalt	
	PF21-01	Grt, Gln, Mca, Qz, Rt, ± Zrc	Blueschist	
	PF21-02	Qz, Rt	Qz-vein	
Monviso	MVE2	Omp, Grt, Qz, Amp, Mca, ± Rt, ± Zrc	Eclogite	1.9 ± 0.2 GPa, 580 ± 40 °C ^(24)^
	MVE4	Omp, Grt, Qz, Amp, Mca, ± Rt, ± Zrc	Eclogite	
	MVE12	Omp, Grt, Qz, Amp, Mca, ± Rt, ± Zrc	Eclogite	
Monte Mucrone	M-E1	Omp, Grt, Gln, Pg, Mca, ± Rt, ± Zrc, ± Qz	Vein in eclogite	1.8 ± 0.1 GPa, 550 ± 50 °C ^(27)^
	M-E2	Omp, Grt, Gln, Pg, Mca, ± Rt, ± Zrc, ± Qz	Vein in eclogite	
	M-Q1	Omp, Grt, Gln, Pg, Mca, Qz ± Rt, ± Zrc	Vein in eclogite	
	M-Q2	Omp, Grt, Gln, Pg, Mca, Qz, ± Rt	Vein in eclogite	
	M-Q3	Omp, Grt, Gln, Pg, Mca, ± Rt, ± Zrc	Vein in eclogite	
Ivrea	IV16-04	Qz, Grt, Kfs, Rt, ± Mca	Metapelite	0.8 ± 0.1 GPa, 770 ± 30 °C ^(8)^
	IV17-05	Grt, Kfs, Qz, Sil, Rt	Metapelite	0.8 ± 0.1 GPa, 915 ± 15 °C ^(8)^
Central Alps				
Alpe Arami	AA16-11	Grt, Omp, Rt, ± Zrc	Eclogite	3.0 ± 0.3 GPa, 830 ± 25 °C ^(26)^
	AA20-1	Grt, Omp, Ky, Qz, Amp, Rt, ± Zrc	Eclogite	
	AA21-01	Grt, Omp, Rt, ± Amp, ± Zrc	Rodingite	
	AA21-02	Grt, Omp, Rt, ± Zrc	Eclogite	
Cima di Gagnone	CdG19/36	Grt, Mca, Qz, Ky, Pl, Bt ± Rt, ± Omp, ± Zrc, ± Ep	Metapelite	2.7 ± 0.1 GPa, 800 ± 50 °C ^(21)^
Alpe Capoli	CP16-03B	Rt, ± Omp, ± Mca, ± Zrc	Rutile vein	2.5 ± 0.3 GPa, 750 ± 75 °C ^(7)^
	CP16-04	Grt, Omp, Qz, Mca, Amp, Rt, ± Zrc	Eclogite	
	CP18-03A	Grt, Omp, Qz, Mca, Amp, Rt, ± Zrc	Eclogite	
	CP18-03B	Grt, Omp, Qz, Mca, Amp, Rt, ± Zrc	Eclogite	
Val Cama	CM20-2	Amp, Grt, Pl, Spl, Rt, Mag, Ttn, Rt, Czo, ± Zrc	Amphibolite	1.2 ± 0.2 GPa, 690 ± 40 °C ^(7)^
	CM20-6	Grt, Cpx, Amp, Rt Ep, Ttn, Ilm, ± Zrc	Metarodingite	
Alpe Senevedo Superiore	AS19-1	Grt, Qz, Mca, Rt, ± Ap, ± Zrc	Metapelite	1.0 ± 0.3 GPa, 475 ± 25 °C ^(3)^
	AS19-2	Grt, Mca, Chl, Amp, Rt, ± Ap, ± Zrc	Metapelite	
	AS19-3	Grt, Qz, Mca, Chl, Amp, Rt, ± Ap, ± Zrc	Metapelite	
Campolungo	19-JH-03	Qz, Mca, Bt, Pl, Grt, Rt, ± Aln, ± Zrn	Metapelite	0.8 ± 0.1 GPa, 600 ± 25 °C ^(4)^
Eastern Alps				
Koralpe	Kor3	Grt, Qz, Pl, Mca, Amp, Bt, Rt, ± Zrc	Metapelite	2.2 ± 0.2 GPa, 685 ± 55 °C ^(25)^
Saualpe	Sau2	Grt, Omp, Ky, Qz, Amp, Rt, ± Ap, ± Zrc	Eclogite	2.2 ± 0.2 GPa 685 ± 55 °C ^(25)^
	Sau3	Grt, Omp, Qz, Mca, Rt, ± Ap, ± Zrc	Eclogite	
Ulten Zone	Ulten	Grt, Ky, Pl, Qz, Rt	Granulite	1.0 ± 0.1 GPa, 660 ± 60 °C ^(5)^
Bohemian Massif				
Sudetes	WPT-4–1	Grt, Qz, Kfs, Pl, ± Rt	Granulite	1.9 ± 0.1 GPa, 950 ± 50 °C ^(1, 19)^
	WPT-4-2B	Grt, Qz, Kfs, Pl, ± Amp, ± Rt	Granulite	
	WPT-10	Grt, Qz, Kfs, Ky, Bt, Pl, ± Rt	Granulite	
Erzgebirge	ERZF5b	Grt, Omp, Qz, Amp, Bt, Rt, ± Zrc	Eclogite	1.8 ± 0.4 GPa, 1050 ± 50 °C ^(20)^
West Gondwana Orogen, Mali	S-508	Qz, ± Rt, ± Zrc	Quartzite	3.3 ± 0.1 GPa, 780 ± 40 °C ^(9)^
	S-520	Qz, ± Rt, ± Zrc	Quartzite	
Western Gneiss Region, Norway	WG17-01	Qz, Grt, Ky, Bt Pl, Sil, Rt, ± Mca, ± Ap, ± Zrc	Metapelite	3.2 ± 0.1 GPa, 850 ± 50 °C ^(6, 12, 17)^
	FJ-RT	Grt, Qz, Ky, Bt, Kfs, Mca, Sil, Rt, ± Mnz, ± Zrc, ± Ap	Metapelite	
Serre Massif, Sardinia, Italy	KINZ	Grt, Crd, Sil, Bt, Pl, ± Rt	Granulite	0.7 ± 0.1 GPa, 800 ± 50 °C ^(23)^
Syros, Cyclades, Greece	SY21-31	Grt, Omp, Gln, ± Cal, ± Qz, ± Ap, ± Ep, ± Rt, ± Zrc	Eclogite-blueschist	2.0 ± 0.1 GPa, 530 ± 17 °C ^(14)^
	SY-KM1	Rt, ± Amp, ± Qz	Placer	
	SY-KM2	Rt	Eclogite	
Eastern Ghats, India	An-1	Qz, Crd, Opx, Spl, Rt	Granulite	0.9 ± 0.1 GPa, 1000 ± 50 °C ^(18)^
	An-2	Qz, Crd, Opx, Spl, Rt	Granulite	
Dabie Shan, China	DB1	Grt, Qz, Omp, Amp, Rt, ± Ky, ± Zrc	Eclogite	3.9 ± 0.1 GPa, 825 ± 25 °C ^(16)^
	DB2	Grt, Qz, Amp, Rt, Bt, ± Zrc	Eclogite	
	SH02-3	Grt, Qz, Omp, Amp, Rt	Eclogite	
	DB6	Cpx, Cc, Grt, Qz, Ky, Sil, Amp, Rt, Ph	Impure marble	4.4 ± 0.4 GPa, 725 ± 35 °C ^(15)^
	DB7	Omp, Grt, Amp, Cal, Chl, Rt	Eclogite	

References: (1) Anczkiewicz et al. (2007), (2) Barnicoat and Fry (1986), (3) Bissig and Hermann (1999), (4) Boston et al. (2017), (5) Braga et al. (2007), (6) Butler et al. (2015), (7) Dale and Holland (2003), (8) Ewing et al. (2013), (9) Ganade et al. (2023), (10) Gauthiez-Putallaz et al. (2016), (11) Groppo et al. (2009), (12) Hacker et al. (2015), (13) Hermann (2003), (14) Laurent et al. (2018), (15) Liu et al. (2014), (16) Liu et al. (2020), (17) March et al. (2022), (18) Mukhopadhyay and Basak (2009), (19) O’Brien et al. (1997), (20) O’Brien and Rötzler (2003), (21) Piccoli et al. (2021), (22) Reinecke (1998), (23) Schenk (1984), (24) Schwartz et al. (2000), (25) Thöni et al. (2008), (26) Trommsdorff et al. (2000), (27) Vho et al. (2020). Mineral abbreviations after Warr (2021)

For *‘cold’ thermal gradients* (~ 150–350 °C/GPa; similar to Honshu and Nicaragua in model D80 of Syracuse et al. (2010)) LT eclogite facies and UHP samples were chosen (‘cold subduction samples’). We selected a large set of mafic samples, as mafic lithologies usually record evidence of cold thermal conditions during subduction. Mafic samples include LT eclogites from the Western Alps (Pfulwe: PF18-14, PF18-25c, PF18-26b, PF18-TB01, PF21-01; Monviso: MVE2, MVE4, MVE12; Lago di Cignana: L31), Eastern Alps (Saualpe: Sau2, Sau3), and Syros, Cyclades, Greece (SY21-31, SY-KM2). Additionally, we selected rutile samples from quartz veins within mafic LT eclogites from two localities in the Western Alps (Pfulwe: PF21-02; Monte Mucrone: M-E1, M-E2, M-Q1, M-Q2, M-Q3). Mafic UHP samples come from Dora-Maira, Western Alps (DM9, DM30) and Dabie Shan, China (DB1, DB2, DB7, SH02-3). Felsic samples were also selected from the localities in Syros and Dora-Maira (Syros: SY-KM1; Dora-Maira: DM2, DM5, DM10), including one sample from a Dora-Maira whiteschist (DM1c) as example of a high-Al–high-Mg lithology, and a metamarl from Dabie Shan (DB6). Additionally, we investigated one metapelite sample from Koralpe, Eastern Alps (Kor3) and two quartzite samples from the West Gondwana Orogen, Mali (S508, S520).

*‘Warm’ thermal gradients* (~ 275–575 °C/GPa; similar to e.g. Cascadia in model D80 of Syracuse et al. (2010)) are represented by pressure-dominated amphibolite facies samples, HT–HP eclogite facies samples, and HT–UHP samples (‘warm subduction samples’). Eight mafic eclogite samples from two localities in the Central Alps were selected (Alpe Arami: AA16-11, AA20-1, AA21-02; Alpe Capoli: CP18-04, CP18-03A, CP18-03B). From the same localities we also investigated a metarodingite (AA21-01) and a rutile sample from a massive rutile vein within an eclogite (CP16-03B). Amphibolite samples were chosen from Val Cama, Central Alps (CM20-2, CM20-6). Felsic samples come from HT–HP eclogite localities in the Central Alps (Campolungo: 19-JH-03; Cima di Gagnone: CdG19/36), from a HP upper greenschist facies locality in the Central Alps (Val Malenco: AS19-1, AS19-2, AS19-3), and from an UHP terrain in the Western Gneiss Region, Norway (WG17-01, FJ-RT). For ‘warm’ subduction settings both mafic and felsic samples were selected to reflect the subduction of both oceanic crust and sediments or continental crust. Even though warm thermal gradients could also represent collisional settings, no collision related samples were chosen.

Most samples from *‘hot’ thermal gradients* (~ 500–1500 °C/GPa) are felsic, as they represent continental metamorphism. We selected granulites from the Italian Alps (Ivrea: IV16-24, IV17-05; Ulten Zone: Ulten), and the Serre Massif, Sardinia, Italy (KINZ), as well as HP granulites from the Sudetes, Bohemian Massif, Poland (WPT4-1, WPT4-2B, WPT10), and the Eastern Ghats, India (AN-1, AN-2). Additionally, one mafic HP granulite sample from Erzgebirge, Bohemian Massif, Germany (ERZF5b) was studied.

Hydrothermal and pegmatitic rutile samples (Table 2) were also analysed to evaluate the potential bias they can cause in investigations of detrital rutile. Five *hydrothermal* samples (6059, A6758, A7653, Rt1, Binn) from different localities within the Central Alps as well as six rutile samples from different *pegmatites* (A5088, A5732, CYW2320, SFJ, GrB, MP2) were analysed.

**Table 2:** Hydrothermal and pegmatitic samples with localities and approximate grain sizes

Locality	Sample	Lithology	grain size
Binntal, Central Alps	Rt1	Hydrothermal	2 cm, elongated
	Binn		2 cm, elongated
Campo Tencia, Central Alps	6059		2 cm, elongated
Faulhorn, Central Alps	A7653		2 cm, elongated
Mompe medel, Central Alps	A6758		0.7 cm, elongated
Alinci, North Macedonia	MP2	Pegmatitic	2 × 5 × 1.5 cm
Bahia, Brazil	GrB		0.1 cm, needles
Brazil	SFJ		2 cm, elongated
Graves Mountain, Georgia, USA	CYW2320		5 cm, rounded
Iragna, Central Alps	A5732		1 cm, elongated
Sobĕslav, Bohemia, Czech Republic	A5088		2 × 2 × 2.5 cm

### Sample preparation

Metamorphic samples (with the exception of the samples 19-JH-03, Wg17-05, FJ-RT, SY21-31, SY-KM2, PF21-02, and samples from Monte Mucrone) were prepared as double polished thick sections, polished to a grade of 3 µm. The samples were removed from their glass plates for FTIR measurements by leaving them in acetone for 8–48 h. Sample thickness lies between 80–130 µm and was determined using a vertically mounted mechanical Mitutoyo ID-S112X micrometre with a precision of ± 3 µm. For LA–ICP–MS measurements, samples were re-glued to glass plates.

The samples from Campolungo, Central Alps (19-JH-03) and the West Gondwana Orogen (S-508, S-510, S-520) were mineral separates. Grains of 20–200 µm size were picked and left un-polished. Grain thickness was determined from the z-position of the calibrated stage during FTIR measurements, with a precision of ± 3 μm. For LA–ICP–MS measurements, grains were mounted in 1-inch epoxy mounts and polished on one side to a grade of 1 µm using grinding paper and diamond polishing paste.

For the large hydrothermal and pegmatitic samples (6059, A5088, A6758, CYW2320, SFJ) approximately 3 mm sized pieces of samples were crushed using an agate mortar to a grain size of 20–200 µm, samples were left un-polished for FTIR analyses and prepared as 1-inch polished epoxy mounts for LA–ICP–MS analyses. Grain thickness was determined during FTIR measurements from the z-stage position.

The single grain samples from Syros (SY21-31, SY-KM2) and Monte Mucrone (M-E1, M-E2, M-Q1, M-Q2, M-Q3), as well as samples Rt1, CYW2320, SFJ, MP2, GrB, Binn, and A5732 were prepared as doubly polished, 1-inch epoxy mounts with a thickness of 120–250 µm. Sample thickness was determined using a mechanical micrometer after the samples were removed from their glass plates and the epoxy was dissolved by leaving them in acetone for ~ 12–48 h. Samples were re-glued on a glass plate for LA–ICP–MS measurements.

Samples PF21-02 and A7653 were prepared as oriented grains. The grains are elongated along their crystallographic c-axis, allowing orientation according to this morphological characteristic. The samples were then cut approximately perpendicular to the crystallographic c-axis. Sample PF21-02 was additionally cut parallel to the c-axis. The resulting cross-sections were mounted in 1-inch epoxy mounts and doubly polished to a thickness of ~ 100–150 µm. Samples were removed from their glass plates by leaving them in acetone for ~ 12 h and re-glued on glass plates for LA–ICP–MS measurements.

### Fourier transform infrared (FTIR) spectroscopy for H_2_O analyses

We performed polarized transmission FTIR spectroscopy at the University of Bern, following the measurement protocol and quantification described in Lueder et al. (2023). Selected grains were mapped to evaluate intra-grain variability of H_2_O contents and H^+^ diffusion. Detailed FTIR maps were measured using the focal plane array (FPA) detector, composed of 64 × 64 liquid nitrogen cooled MCT elements on a square array. To improve the quality of measured spectra, maps were collected with a binning of 2, resulting in a spatial resolution of 5.4 × 5.4 µm. Data was acquired in a wavenumber range of 900–3800 cm^−1^, with a resolution of 8 cm^−1^ and 64 scans. Maps were baseline corrected and corrected for atmospheric interference within the OPUS© software.

Calculations of H_2_O contents and evaluation of FTIR maps was done using the software *SpecXY* (Gies et al. 2023). Using the classification module of *SpecMaps*, fractures, mineral inclusions and surrounding phases were excluded from the data evaluation. To identify different OH-band positions related to different cations, we applied a deconvolution to the OH-range of each individual spectrum from single spot analyses and maps. The peak heights and width of fixed position Lorentzian peaks were fitted to trace-element dependent OH band positions at 3280 cm^−1^ for Ti^3+^, 3295 cm^−1^ for Fe^3+^, 3323 cm^−1^ for Al^3+^, 3350 cm^−1^ for Mg^2+^, 3370 cm^−1^ for Fe^2+^, and 3390 cm^−1^ for Cr^2+^ (Lueder et al. 2023). OH bands related to Cr^3+^ were described for synthetic rutile (e.g. Bromiley and Hilairet 2005), but are not yet observed in natural rutile. The integration range within the OH-region (3200–3500 cm^−1^) was chosen depending on the observed OH-bands. For samples showing only OH-bands related to Ti^3+^, Fe^3+^, and/or Al^3+^ an integration range between 3200–3400 cm^−1^ was chosen, for samples showing additional OH-bands related to Mg^2+^ and/or Fe^2+^ an integration range between 3200–3450 cm^−1^, and for samples showing a Cr^2+^-related OH-band an integration range between 3200–3550 cm^−1^. Additionally, trace-element dependent H_2_O contents were calculated from deconvoluted spectra in the same integration ranges as specified above.

### LA–ICP–MS for trace-element analyses

LA–ICP–MS analysis on the same grains previously measured by FTIR was performed at the University of Bern. A Resonetics RESOlution SE 193 nm excimer laser system equipped with a S-155 large volume constant geometry ablation cell (Laurin Technic, Australia) coupled to an Agilent 7900 quadrupole ICP–MS system was used. Ablation was performed in an ultra-pure He (0.04 l min^−1^) and N_2_ (0.003 l min^−1^) mix with Ar (0.86 l min^−1^) immediately after the ablation cell. A surface energy density on the sample of 4 J/cm^2^ and a laser repetition rate of 5 Hz were used. The beam size was chosen between 14 and 50 µm, depending on grain size. A pre-ablation of three pulses with a slightly larger beam size was performed to clean the surface of the sample. A suite of 26 elements was measured over a total acquisition time of ~ 85 s per measurement, with ~ 50 s gas background, ~ 5 s pre-ablation, and ~ 30 s sample signal. Primary- (SRM-NIST610) and secondary standards (SRM-NIST612) were measured every 10–30 min (Jochum et al. 2011). For data reduction, the software Iolite was used (Hellstrom et al. 2008; Paton et al. 2011). A polynomial function was fitted to the standard measurements to correct for instrument drift, and backgrounds were subtracted from all measurements using a step-forward function. All samples were quantified using a fixed value of 59.94 wt% Ti.

## Results

### Trace-element contents

**Fig. 2:**
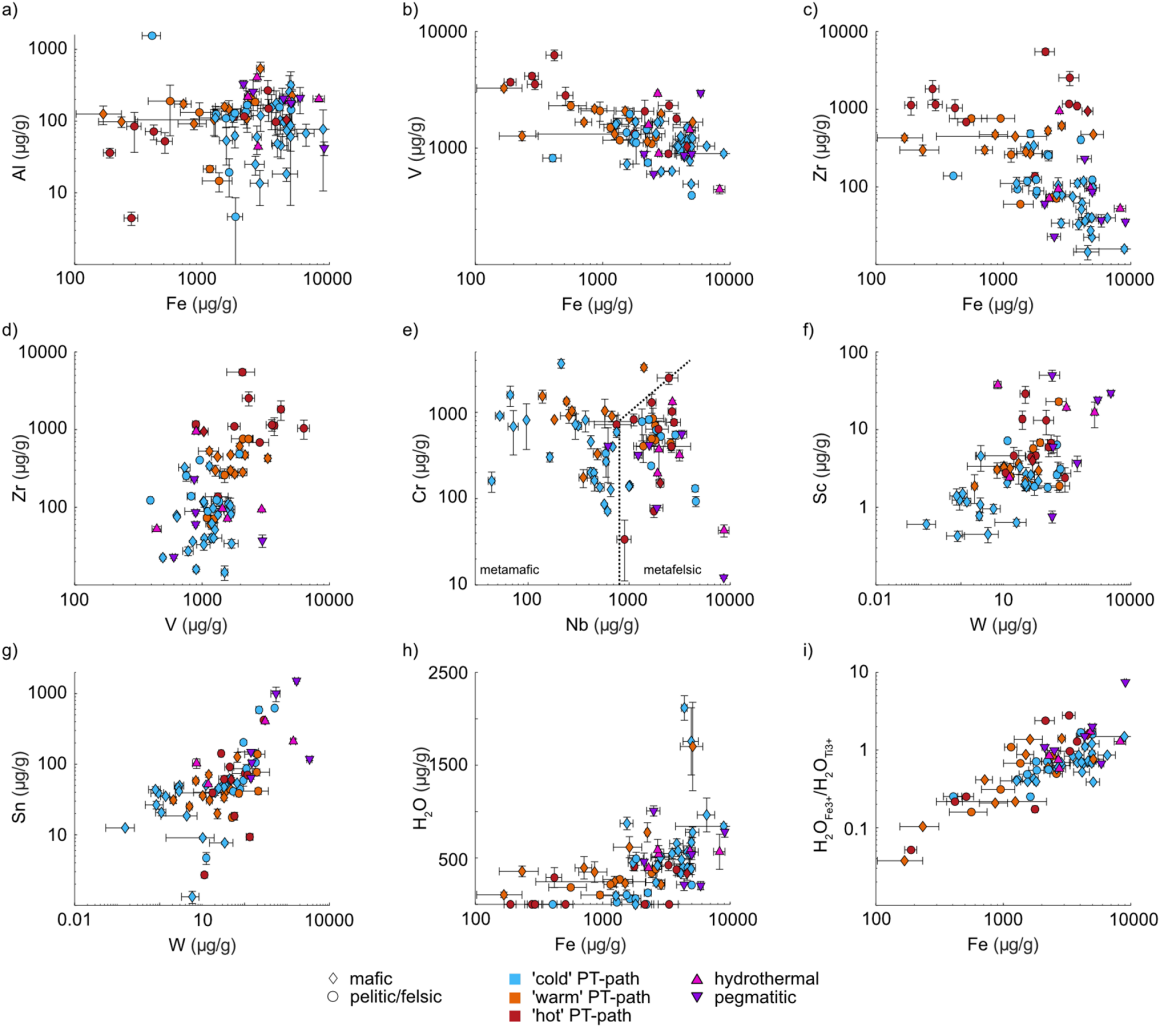
Trace-element and H_2_O contents for the analysed rutile grains. **a** Al vs. Fe. **b** V vs. Fe. **c** Zr vs. Fe. **d** Zr vs. V. and **e** Cr. Vs. Nb, with the boundary between mafic and felsic according to Meinhold et al. (2008). **f** Sc vs. W. **g** Sn vs. W. **h** Total H_2_O vs. Fe. **i** H_2_O_[Fe3+]_/H_2_O_[Ti3+]_ vs. total Fe, with H_2_O_[Fe3+]_ and H_2_O_[Ti3+]_ being the Fe^3+^- and Ti^3+^-related H_2_O contents, respectively. Mafic samples are shown as diamonds, felsic samples as circles. Blue symbols indicate ‘cold’ subduction thermal gradients (~ 150–350 °C/GPa), orange ‘warm’ thermal gradients (~ 275–575 °C/GPa) and red ‘hot’ thermal gradients (~ 500–1500 °C/GPa). Hydrothermal and pegmatitic samples are depicted as pink and purple triangles respectively. Symbols represent median values for samples with median absolute deviations given as uncertainties

The observed trace-element contents in the analysed rutile samples are highly variable (Online Resource 2). Aluminium contents range between ~ 5–500 μg/g in metamorphic rutile, with no significant differences between different P–T conditions or lithologies (Fig. 2a). Hydrothermal and pegmatitic samples have generally higher Al contents > 200 μg/g, but still within the range observed for metamorphic rutile. Unusually high Al contents are observed in the sample from the Dora-Maira whiteschist at ~ 1550 μg/g.

Iron contents range between ~ 200–10,000 μg/g (Fig. 2a–c). The lowest Fe contents (< 500 μg/g) are observed in LP, ‘hot’ geotherm samples. ‘Warm’ subduction samples have comparably higher Fe contents of ~ 500–3000 μg/g. The highest Fe contents are observed in ‘cold’ subduction samples at ~ 1000–10,000 μg/g. HP ‘hot’ geotherm samples overlap with ‘warm’ and ‘cold’ subduction samples in a range of ~ 2000–4000 μg/g Fe. There is no clear difference in Fe contents depending on lithology of metamorphic samples. Hydrothermal and pegmatitic rutile have high Fe contents in a similar range as ‘cold’ subduction samples (~ 2000–10,000 μg/g).

Vanadium contents are negatively correlated with Fe contents (Fig. 2b). ‘Cold’ subduction samples have moderate V contents of ~ 400–2000 μg/g. ‘Warm’ subduction samples and HP, ‘hot’ geotherm samples have mostly higher V contents of ~ 1000–3000 μg/g. The highest V contents are observed in LP, ‘hot’ geotherm samples (~ 3000–6000 μg/g). As for Al, neither V nor Fe contents vary with lithology. Hydrothermal and pegmatitic rutile has highly variable V contents, spanning almost the same range as metamorphic samples.

Zirconium contents vary between ~ 10–10,000 μg/g and clearly increase from ‘cold’ towards ‘warm’ and ‘hot’ geotherm samples (Fig. 2c). Zirconium and Fe are negatively correlated, with low Fe–high Zr in LP, ‘hot’ geotherm samples, intermediated Fe and Zr contents in ‘warm’ and HP, granulite facies samples, and high Fe–low Zr in ‘cold’ subduction samples (Fig. 2c). Few ‘hot’ geotherm, felsic samples do not follow this general correlation and show elevated Fe contents relative to other samples with comparable Zr contents. Contrary, Zr and V are positively correlated with both Zr and V contents increasing from ‘cold’ subduction samples, to ‘warm’ and HP granulite facies samples, to LP ‘hot’ geotherm samples (Fig. 2d). For both Zr–Fe and Zr–V, hydrothermal and pegmatitic samples overlap with ‘cold’ subduction samples. As Zr content in rutile is a thermometer (e.g. Zack et al. 2004a), the increasing V and decreasing Fe contents might also be temperature dependent.

Chromium contents vary between ~ 100–3000 μg/g (Fig. 2e). Generally, ‘cold’ subduction samples have slightly lower Cr contents than ‘warm’ and ‘hot’ geotherm samples. However, this difference is not as strongly pronounced for mafic samples and almost disappears for felsic samples. Most hydrothermal and pegmatitic rutile has Cr contents of ~ 200–1000 μg/g, with few samples showing very low Cr contents < 100 μg/g.

The Nb content distinguishes between mafic and felsic samples. Almost all mafic samples have Nb contents < 800 μg/g, whereas rutile from felsic lithologies has Nb contents of ~ 800–5000 μg/g (Fig. 2e). No significant variations of Nb contents with metamorphic P–T conditions can be identified. Hydrothermal and pegmatitic samples have high Nb contents, similar to felsic metamorphic rutiles, of ~ 1000–10,000 μg/g. Most samples follow the classification of Meinhold et al. (2008) for the differentiation of mafic and felsic rutile (i.e., derived from a felsic host rock) based on their Cr and Nb contents, with hydrothermal and pegmatitic samples also falling into the field for felsic rutile (Fig. 2e).

Tungsten contents are highly variable at up to ~ 300 μg/g. Generally, mafic samples have lower W contents than felsic samples, and ‘cold’ subduction samples have lower W contents compared to ‘warm’ and ‘hot’ geotherm samples, however, there is significant overlap amongst the different lithologies and P–T conditions (Fig. 2f, g). Hydrothermal and pegmatitic samples show an even more pronounced spread in W contents (~ 10–10,000 μg/g). Even though W content in hydrothermal and pegmatitic rutile tends to be higher, there is still a significant overlap with metamorphic rutile.

Scandium contents are generally low in all samples (Fig. 2f). ‘Cold’ subduction samples have the lowest Sc contents (< 6 μg/g), with slightly higher Sc in felsic compared to mafic samples. ‘Warm’ subduction samples show slightly higher Sc contents (~ 3–10 μg/g) than ‘cold’ subduction samples, and the highest Sc contents can be observed in ‘hot’ geotherm samples (~ 5–30 μg/g). Hydrothermal and pegmatitic rutile show a large spread in Sc content of < 1–60 μg/g. A general correlation between Sc and W contents can be observed, with both Sc and W increasing from ‘cold’ to ‘warm’ to ‘hot’ geotherm samples to hydrothermal and pegmatitic rutile.

Tin contents differ between felsic and mafic samples (Fig. 2g). Mafic samples have Sn contents of ~ 10–70 μg/g, independent of P–T conditions. Felsic samples show a significantly larger spread in Sn contents of up to 200 μg/g, also without significant difference between P–T conditions. Hydrothermal and pegmatitic samples have higher Sn contents, above those observed for mafic samples, at ~ 70–2000 μg/g. A clear correlation between Sn and W can be observed (Fig. 2g). Both Sn and W contents increase from mafic ‘cold’ subduction samples, to felsic ‘cold’ subduction samples, to ‘warm’ subduction samples, to ‘hot’ geotherm samples, and to hydrothermal and pegmatitic samples.

### Types of FTIR spectra

Peak deconvolution is used to identify different defects contributing to the OH-bands of the FTIR signal. Lueder et al. (2023) identified six different trace-element related H^+^-defects in natural rutile: three narrow bands at ~ 3278 cm^−1^, ~ 3295 cm^−1^, and ~ 3323 cm^−1^ related to Ti^3+^, Fe^3+^ and Al^3+^, respectively and three wide bands at ~ 3345 cm^−1^, ~ 3370 cm^−1^, and ~ 3390 cm^−1^, related to Mg^2+^, Fe^2+^, and Cr^2+^, respectively. Based on these observations we performed peak deconvolution on all measured spectra.

**Fig. 3:**
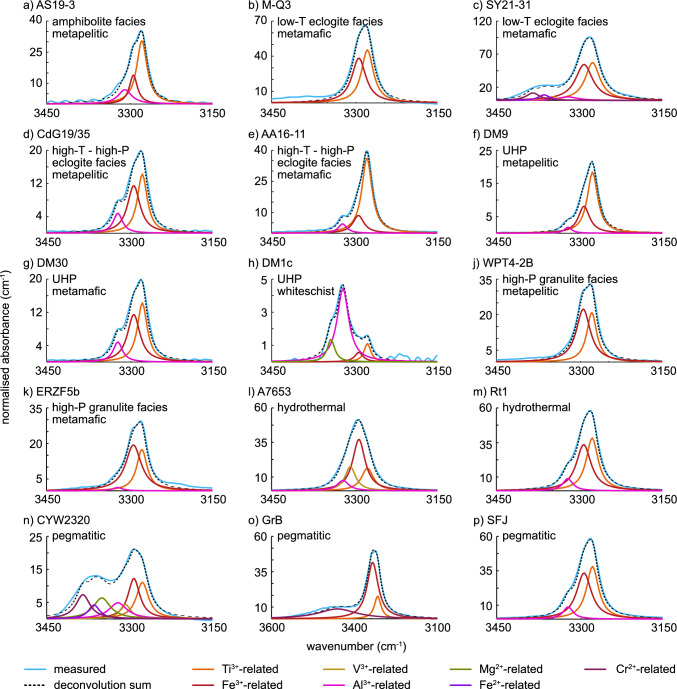
Deconvolutions of average spectra of representative rutile samples from all investigated P–T–X conditions. Spectra are averaged over all measured spectra for a sample. Sample AS19-3 is from an amphibolite facies metapelite. Samples M-Q3 and SY21-31 have LT eclogite facies peak conditions, sample SY21-31 is from a mafic rock, and sample M-Q3 is from a quartz vein associated with an eclogite. Samples CdG19/35 and AA16-11 come from HT–HP eclogite facies conditions, sample CdG19/35 is metapelitic, and samples AA16-11 are mafic. Samples DM9, DM30, and DM1c experienced UHP peak metamorphic conditions. Sample DM9 is metapelitic, sample DM30 is mafic and sample DM1c is from an Al- and Mg-rich whiteschist. Samples WPT4-2B and ERZF5b are from HP granulite facies samples. Sample WPT4-2B is metapelitic, sample ERZF5b is mafic. Samples A7653 and Rt1 are hydrothermal rutile. Samples CYW2320, GrB, and SFJ are from different pegmatites

All samples show H^+^-defects related to Ti^3+^ and Fe^3+^. Most samples from Monviso, Pfulwe, Monte Mucrone (Fig. 3b), Ivrea, Western Alps, Saualpe, the Ulten Zone, Eastern Alps, Dabie Shan, the Serre Massif, and the Sudetes (Fig. 3j) do not show any additional H^+^-defects. The most common class of spectra show an additional Al^3+^-related OH-band, which can vary strongly in relative absorbance. In total, 23 samples, from all lithologies and P–T conditions, show a significant contribution of the Al^3+^-related OH-band to the overall signal (e.g., mafic HP ganulite facies: Fig. 3k, felsic HP–HT eclogite: Fig. 3d, mafic HP– HT eclogite Fig. 3e, felsic UHP: Fig. 3f, mafic UHP: Fig. 3g). We observed only minor contributions of the Al^3+^-related OH-bands in two mafic amphibolite facies samples, one mafic LT eclogite facies sample, and three mafic HT–HP eclogite facies samples. Most hydrothermal and pegmatitic samples also fall into the class, showing medium to high contributions of the Al^3+^-related OH-band to the spectrum (Fig. 3m, p).

The third class is defined as spectra with all three previously described OH-bands (Ti^3+^-, Fe^3+^-, Al^3+^-related) and additional OH-bands which are related to divalent cations. Within the class, different groups are identified. The first group is rutile from two LT–HP rocks samples from Pfulwe, which only show a Fe^2+^-related OH-band in addition to the OH-bands related to trivalent cations. The rutile from a Fe-Ti-metagabbro shows significantly less relative absorbance of the Fe^2+^-related OH band than the sample from a quartz-vein associated to an eclogite. One pegmatitic sample shows only an OH-band related to Cr^2+^, other than the Fe^3+^- and Ti^3+^-related OH-bands (Fig. 3o). One sample from a metamorphic placer deposit from Syros has both a Cr^2+^-related OH-band and a Fe^2+^-related OH-band. The absorbance of both the Fe^2+^- and Cr^2+^-related OH-band are comparable. One sample from a vein associated with an eclogite from Alpe Capoli and a sample from an eclogite from Syros have an additional OH-band related to Mg^2+^ (Fig. 3c, d). The OH-bands related to divalent cations in both samples have a much more significant contribution to the overall FTIR signal compared to rutile from the placer deposit from Syros. The eclogite sample from Syros has similar absorbance of all three OH-bands related to divalent cations, whereas the sample from Alpe Capoli has significantly similar absorbance for the OH-bands related to Fe^2+^ and Cr^2+^, and significantly lower absorbance for the Mg^2+^-related OH-band. The investigated sample from a whiteschist from Dora-Maira shows a distinctly different OH-spectrum (Fig. 3h). This sample has an OH-band related to Mg^2+^ in addition to the OH-bands related to trivalent cations. Additionally, for this sample, the Al^3+^-related OH-band is the most dominant, whereas in all other previously described samples the Ti^3+^- and/or Fe^3+^-related OH-bands dominate the spectra.

The last class are rutile spectra that show a previously undescribed OH-band. Two pegmatitic and one hydrothermal sample show an OH-band at ~ 3310 cm^−1^ as shoulder on the main Ti^3+^- and Fe^3+^ related OH bands. The hydrothermal sample only shows OH-bands related to trivalent cations other than the 3310 cm^−1^ band (Fig. 3l), whereas both pegmatitic samples show all OH-bands related to di- and trivalent cations that have been previously described (Fig. 3n).

There is no clear link between spectral classes or subgroups with either peak metamorphic P–T conditions or sample lithology. Most samples, independent of peak metamorphic conditions and bulk rock composition are dominated by OH-bands related to trivalent cations. OH-bands related to divalent cations are observed mainly in pegmatitic and vein-related rutile.

### H_2_O contents

**Fig. 4:**
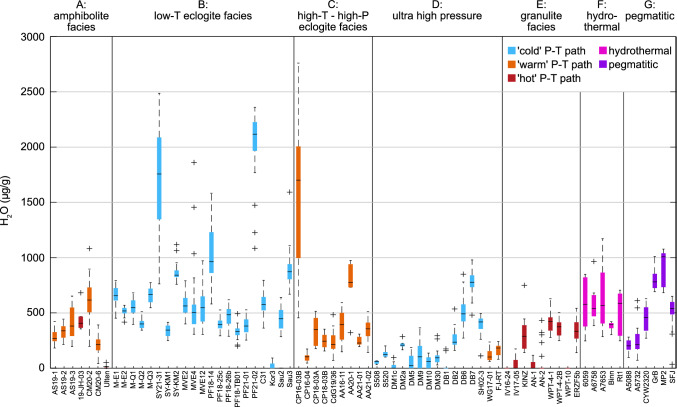
Variability of H_2_O contents in rutile. Black lines indicate the median H_2_O content for each sample (n = 5–50), boxes give the 25th and 75th percentile, black dashed lines indicate the total spread of H_2_O contents, and black crosses depict outliers. Samples are grouped by peak P–T-conditions into amphibolite facies, LT- and HT–HP eclogite facies, UHP and granulite facies, as well as hydrothermal and pegmatitic samples. Box colors indicate subduction thermal gradients, blue represents ‘cold subduction’ (~ 150–350 °C/GPa), orange ‘warm subduction’ (~ 275–575 °C/GPa), and red ‘hot subduction’ (~ 500–1500 °C/GPa). Hydrothermal samples are shown in pink and pegmatitic samples in purple

Total and trace-element dependent H_2_O contents have been calculated from deconvoluted spectra according to Eq. (6) in Lueder et al. 2023. Observed H_2_O contents vary strongly from nominally dry (H_2_O below detection limit of ~ 10 μg/g) up to ~ 2700 μg/g in individual grains (Fig. 4, see also online resource 2). H_2_O contents are discussed in terms of the samples peak metamorphic facies (i.e., amphibolite, LT eclogite) and the P/T gradient of their peak conditions (i.e., ‘warm’ PT-path, ‘hot’ PT-path, Fig. 4). Amphibolite facies samples are related to ‘warm’ and ‘hot’ thermal gradients, LT eclogite facies samples are related to ‘cold’ thermal gradients, HT–HP eclogite facies samples are related to ‘warm’ thermal gradients, UHP samples are mainly related to ‘warm’ thermal gradients, and granulite facies samples are related to ‘hot’ thermal gradients.

Rutile grains from* amphibolite facies* (Fig. 4, type A) metapelite samples have H_2_O contents of ~ 200–600 μg/g, with an inter grain variability (25th–75th percentile) of ~ 100–300 μg/g. Trace-element related H_2_O contents vary between the localities of the Central Alps. Four samples show most of the H_2_O related to Ti^3+^, with little H_2_O related to Fe^3+^, whereas two samples have ~ 2/3 Fe^3+^-related H_2_O. There is no observable difference between the ‘hot’ PT-path sample and the ‘warm’ PT-path samples.

Generally, H_2_O contents in rutile from *LT eclogite facies* (Fig. 4, type B) samples are higher at ~ 350–700 μg/g in most samples. Additionally, the inter grain variability is lower at ~ 50–200 μg/g. Significantly higher H_2_O contents are observed in five LT eclogite facies samples with ~ 850–2100 μg/g. The inter grain variability for the samples with the highest H_2_O contents is also significantly higher at mostly ~ 300–400 μg/g and up to ~ 2500 μg/g H_2_O content in individual grains. One metapelite sample from a LT sample shows mostly nominally dry grains with few grains reaching up to ~ 70 μg/g. Generally, Ti^3+^-related H_2_O contents are highest with ~ 50–70% of the total H_2_O related to Ti^3+^. Most samples also have high contents of Fe^3+^-related H_2_O. In the samples with very high H_2_O contents, H_2_O related to divalent cations has a significant contribution to the total H_2_O content.

Rutile in *HT–HP eclogite facies* (Fig. 4, type C) samples have lower H_2_O contents compared to LT eclogite facies samples at ~ 100–400 μg/g, thus largely overlap with amphibolite facies samples. Two samples from the Central Alps have significantly higher H_2_O contents of ~ 800–1400 μg/g and up to ~ 2700 μg/g in cores of individual grains. The intra grain variability of H_2_O contents is generally high at ~ 150–300 μg/g and can reach up to ~ 1000 μg/g for the vein-related sample with high median H_2_O contens. H_2_O contents related to Ti^3+^ are highest, with ~ 65–90% of the total H_2_O related to Ti^3+^ in most samples. In samples from relatively lower temperatures, Fe^3+^-related H_2_O contents can also be relatively high. One sample with high H_2_O contents also shows ~ 10% Cr^2+^-related H_2_O.

Most *UHP* samples have rutile with low H_2_O of < 200 μg/g (Fig. 4, type D). Two samples are nominally dry (H_2_O-content below detection limit of ~ 10 μg/g) and many H_2_O -bearing samples contain nominally dry grains. Inter grain variabilities for metapelitic samples is higher than for mafic samples at ~ 200–300 μg/g and ~ 50 μg/g, respectively. Approximately 65–75% of H_2_O is related to Ti^3+^, with low Fe^3+^-related H_2_O contents.

Rutile in LP *granulite facies* samples are mostly nominally dry (Fig. 4, type E) with the exception of one sample that has H_2_O contents of ~ 300 μg/g. The H_2_O content of HP granulite facies rutile is significantly higher at ~ 300–400 μg/g with high inter grain variabilities of ~ 200–300 μg/g. In LP granulite facies samples, Ti^3+^-related H_2_O contents of rutile are higher than Fe^3+^-related H_2_O contents, whereas rutile in HP granulite facies samples has higher Fe^3+^-related H_2_O contents than Ti^3+^-related H_2_O contents.

*Hydrothermal* (Fig. 4, type F) *and pegmatitic* (Fig. 4, type G) rutile samples have high H_2_O contents, in a similar range as LT eclogite facies samples. For most samples, H_2_O contents range between ~ 400–1000 μg/g with high inter/intra grain variabilities of ~ 300–500 μg/g. Trace-element related H_2_O contents vary strongly for hydrothermal and pegmatitic samples and no systematics can be identified within the investigated samples.

Generally, Ti^3+^- and Fe^3+^-related defects are the most dominant (see online resource 2). However, the absorbance ratios between H^+^ related to Fe^3+^ over H^+^ related to Ti^3+^ (${\text{H}}^{ + }_{{{\text{Fe}}^{3 + } }} /{\text{H}}^{ + }_{{{\text{Ti}}^{3 + } }}$) vary broadly between ~ 0.1–26.6. Most samples have a ratio below 1, thus H^+^ is preferentially coupled with Ti^3+^ over Fe^3+^. The samples from Val Cama and Cima di Gagnone, all samples from Syros, the vein-related sample from Pfulwe, the HP granulite facies samples, and half of the hydrothermal and pegmatitic samples have ratios above 1, meaning that H^+^ is preferentially coupled with Fe^3+^ instead of Ti^3+^. The ${\text{H}}^{ + }_{{{\text{Ti}}^{3 + } }} /{\text{H}}^{ + }_{{{\text{Fe}}^{3 + } }}$-ratio does not correlate with the overall H_2_O content. However, a clear correlation of both total H_2_O content and ${\text{H}}^{ + }_{{{\text{Fe}}^{3 + } }} /{\text{H}}^{ + }_{{{\text{Ti}}^{3 + } }}$-ratio with total Fe content is evident (Fig. 2h, i). Generally, samples with high Fe contents have high H_2_O contents and ${\text{H}}^{ + }_{{{\text{Fe}}^{3 + } }} /{\text{H}}^{ + }_{{{\text{Ti}}^{3 + } }}$-ratios. ‘Cold’ subduction related samples with high Fe contents (> 1000 μg/g) generally tend to have high H_2_O contents (> 450 μg/g) and high ${\text{H}}^{ + }_{{{\text{Fe}}^{3 + } }} /{\text{H}}^{ + }_{{{\text{Ti}}^{3 + } }}$-ratios of ~ 0.3–2, whereas ‘warm’ and ‘hot’ geotherm samples with low H_2_O contents (< 500 μg/g) and low ${\text{H}}^{ + }_{{{\text{Fe}}^{3 + } }} /{\text{H}}^{ + }_{{{\text{Ti}}^{3 + } }}$-ratios of ~ 0.04–2 have low Fe contents (< 1000 μg/g). Hydrothermal and pegmatitic rutile overlaps largely with ‘cold’ subduction related samples. The overall systematics of H_2_O vs. Fe (Fig. 2h) appear to oppose Zr vs. Fe trends (Fig. 2c). With increasing Fe contents, Zr decreases and H_2_O increases non-linearly from granulite facies samples, to ‘warm’ subduction samples, to ‘cold’ subduction, hydrothermal and pegmatitic samples.

### FTIR mapping

**Fig. 5:**
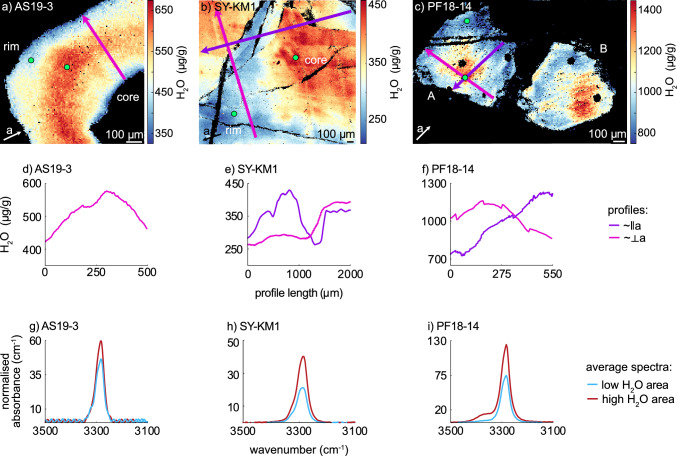
Quantitative H_2_O maps with profiles and representative spectra of mapped rutile grains. **a**–**c** Maps showing total (summed) H_2_O contents in μg/g as obtained from peak deconvolutions. Orientation of the crystallographic a-axis was determined from polarization angles during FTIR analysis and is indicated for each grain. None of the analysed grains have the crystallographic c-axis within the cut plain. Purple and pink arrows indicate locations of transects depicted in **d**–**f**. Purple profiles are taken approximately parallel to the crystallographic a-axis, as has been identified from polarization angles, pink profiles are taken approximately perpendicular to the crystallographic a-axis. Profiles are smoothed using a running average over ~ 5–30 μm to reduce noise. **g**–**i** OH-region of representative spectra extracted from high resolution maps. Blue spectra are from areas with low H_2_O contents close to the rim of each grain, red spectra are from areas with high H_2_O contents closer to the core of each grain. Green spots in **a**–**c** indicate locations of extracted spectra. Spectra were averaged from 5–20 pixels

We performed high resolution FTIR mapping to study the intra grain variability of H_2_O contents. Three maps of representative grains from LT metamorphic samples have been selected (Fig. 5); an amphibolite facies metapelite (AS19-3) and two mafic LT eclogite facies samples (SY-KM1, PF18-14). From all maps, profiles approximately parallel and/or perpendicular to the crystallographic a-axis were extracted. Samples from higher metamorphic grades (granulite facies, UHP) could not be mapped as rutile grains typically were too small.

In the *amphibolite facies* metapelite (Fig. 5a), rutile H_2_O contents increase from ~ 300 μg/g in the core, at the contact to a large inclusion of white mica, to ~ 600 μg/g within the mantle, before decreasing again to ~ 300 μg/g at the rim. An approximately perpendicular profile to the concentric zones shows a continuous slow increase from the core towards the highest H_2_O contents in the mantle and a steeper decrease towards the rim of the grain (Fig. 5d).

*The LT eclogite* from Syros has rutile that shows domain-like zoning with areas of high H_2_O contents up to ~ 400–450 μg/g and areas with low H_2_O contents of ~ 250 μg/g (Fig. 5b). Areas of high H_2_O contents are mostly in the core of rutile grains, whereas areas of low H_2_O contents mainly occur towards rims and large intra-grain fractures. The H_2_O profile approximately perpendicular to the crystallographic a-axis shows a flatter pattern within the area of low H_2_O content and a steep increase toward the area of high H_2_O contents (Fig. 5e). The profile taken approximately parallel to the crystallographic a-axis shows a symmetric increase and decrease from an area of low H_2_O content to high H_2_O content and back to an area of low H_2_O content, as well as a jump in H_2_O content where an area of low H_2_O content and high H_2_O content are separated by a large fracture (Fig. 5e).

The *LT eclogite facies* Fe-Ti-metagabbro from Pfulwe, Central Alps, shows a clear core-rim zoning in two different large rutile grains (Fig. 5c). The highest H_2_O contents in grain A corresponds to the grain centre and reaches ~ 1350 μg/g. Both profiles, approximately parallel and perpendicular to the crystallographic a-axis, show symmetric decreases from the centre towards the rim, with ~ 800 μg/g H_2_O at the rim (Fig. 5f).

Representative spectra have been extracted from cores/areas of high H_2_O content and rims/areas of low H_2_O content for all maps (Fig. 5g–i). Generally, in areas with lower total H_2_O contents, the relative contribution of H_2_O related to divalent cations (Mg^2+^, Fe^2+^, Cr^2+^) to the overall H_2_O content is less than in areas of higher total H_2_O contents. The LT eclogite from Pfulwe (PF18-14, Fig. 5i) has significant H_2_O contents related to divalent cations in areas of high H_2_O content. As their total H_2_O contents decrease to ~ 54% of the highest H_2_O contents, the H_2_O contents related to divalent cations decreases from ~ 10% of the total H_2_O content to less than 1% of the total H_2_O content. For samples only containing H^+^ related to Ti^3+^, Fe^3+^, and Al^3+^, Al^3+^-related H_2_O contents show the least dramatic changes. The Al^3+^-related H_2_O contents are approximately the same in low and high H_2_O content areas or decrease slightly, whereas Ti^3+^ and Fe^3+^-related H_2_O contents in low H_2_O areas can decrease to less than half of the Ti^3+^ and Fe^3+^-related H_2_O contents of high H_2_O areas. However, the decrease in Ti^3+^- and Fe^3+^-related H_2_O contents are not equal, resulting in variable ${\text{H}}^{ + }_{{{\text{Fe}}^{3 + } }} /{\text{H}}^{ + }_{{{\text{Ti}}^{3 + } }}$-ratios. This does not correlate with average ${\text{H}}^{ + }_{{{\text{Fe}}^{3 + } }} /{\text{H}}^{ + }_{{{\text{Ti}}^{3 + } }}$-ratio of a sample, sample lithology, or P–T conditions.

## Discussion

### Applicability of trace-element discrimination diagrams

It has been demonstrated that rutile trace-element geochemistry is dependent on protolith composition (e.g., Clark and Williams-Jones 2004; Zack et al. 2004a, b; Meinhold et al. 2008; Agangi et al. 2019; Pereira et al. 2021). Discrimination diagrams based on rutile chemistry have been developed and are commonly applied in provenance analyses. Previous studies have shown that rutile from hydrothermal veins, felsic magmatic rocks, and ore deposits generally have high W, Sn, Sc, and Sb contents (e.g., Clark and Williams-Jones 2004; Meinhold 2010; Agangi et al. 2019; Pereira et al. 2021). Thus, discrimination diagrams based on these trace elements are used to distinguish metamorphic, magmatic, and hydrothermal rutile, and therefore are also needed to identify ‘cold’ subduction related rutile. However, fields in trace-element discrimination diagrams related to metamorphic and non-metamorphic rutile compositions often overlap (e.g., Agangi et al. 2019; Pereira et al. 2019). In our data set, we see the highest variability in W and Sn between metamorphic and hydrothermal- or pegmatitic samples, whereas Sb and Sc contents are generally similar. All mafic samples have relatively low Sn and W contents below 100 μg/g (Fig. 2e). Hydrothermal and pegmatitic samples have generally high W and/or Sn contents of up to ~ 3400 μg/g W and ~ 1500 μg/g Sn. Felsic samples largely overlap with both mafic and hydrothermal- and pegmatitic rutile.

**Fig. 6:**
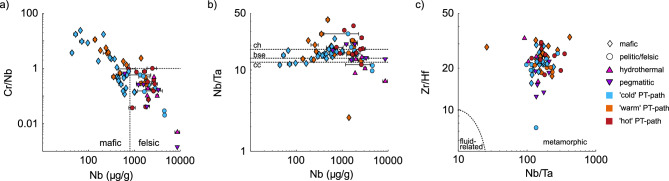
Discrimination diagrams for **a** Cr/Nb vs. Nb with the boundary between mafic and felsic from Meinhold et al. (2008). **b** Nb/Ta vs. Nb as indicator of magmatic source, with approximate values for chondrite (ch), bulk silicate earth (bse), and continental crust (cc). **c** Zr/Hf vs. Nb/Ta, with the field for metamorphic rutile according to Pereira et al. (2021). Mafic samples are shown as diamonds, metapelitic and felsic samples as circles. Blue symbols indicate ‘cold’ subduction thermal gradients (~ 150–350 °C/GPa), orange ‘warm’ thermal gradients (~ 275–575 °C/GPa) and red ‘hot’ thermal gradients (~ 500–1500 °C/GPa). Hydrothermal and pegmatitic samples are depicted as pink and purple triangles respectively. Symbols represent median values for samples with median absolute deviations given as uncertainties (see online resource 2)

Zack et al. (2004b) first proposed a discrimination tool for mafic and felsic rutile based on Cr and Nb content, which was refined by Meinhold et al. (2008). Generally, rutile derived from felsic rocks have high Nb contents (> 800 μg/g) and a negative log(Cr/Nb), whereas rutile from mafic rocks have mostly positive log(Cr/Nb) and/or Nb contents below 800 μg/g. As subducted oceanic crust is often volumetrically dominated by mafic lithologies, many metamorphosed products of subduction of oceanic crust are mafic and, it is therefore important to distinguish mafic and felsic derived rutile. From our data set, most samples follow that discrimination (Fig. 6a), with few exceptions. A sample from a metamarl is not covered by the classification of mafic vs felsic. The rutile grains from quartz veins in an eclogite from Monte Mucrone fall into the felsic field, which may indicate that the vein-forming fluids were at least partly derived from the felsic country rocks surrounding the small eclogitic lenses that host the veins. A mafic HP granulite facies sample from the Erzgebirge, Bohemian Massif has very high Nb contents (2030 ± 56 μg/g) and normal Cr contents (152 ± 17 μg/g), and therefore falls into the felsic field even though it is mafic. Both hydrothermal and pegmatitic rutile are not considered in the classic Nb-Cr discrimination diagrams. The typically high Nb contents in rutile from these rock types results in negative log(Cr/Nb) values. Thus, they fall into the ‘felsic’ field, with the exception of sample A5088. Consequently, in a detrital dataset, hydrothermal and pegmatitic rutile potentially can be incorrectly grouped with rutile from felsic metamorphic rocks, whereas mafic samples with positive log(Cr/Nb) values or Nb contents below 800 μg/g can be well distinguished from rutile from other lithologies.

Rutile is the main host of HFSE in mafic rocks and controls the Nb–Ta budget of the bulk rock (e.g., Hermann 2002; Zack et al. 2002). Thus, Nb/Ta-ratios in rutile can be used to infer magmatic- and subduction zone processes (e.g., Foley et al. 2000; Rudnick et al. 2000; Stepanov and Hermann 2013). Most studied samples have subchondritic Nb/Ta-ratios of ~ 10–18 (Fig. 6b), thus are depleted in Nb in relation to Ta, as is typical for rutile from subducted oceanic- and continental crust (e.g., Meinhold 2010). Combining Nb/Ta-ratios and Zr/Hf-ratios has been used to differentiate metamorphic rutile and rutile related to LP fluid or magmatic sources (e.g. Pereira et al. 2021). Rutile with high Nb/Ta-ratios > 100 have been interpreted to be magmatic and low Zr/Hf- and Nb/Ta-ratios have been associated with fluid-related rutile, whereas Zr/Hf-ratios of ~ 10–60 and Nb/Ta-ratios of ~ 5–100 have been attributed to metamorphic rutile. Almost all rutile samples investigated in this study fall into the field of metamorphic rutile (Fig. 6c), with the exception of one eclogite and the placer deposit from Syros. Thus, none of the investigated hydrothermal and pegmatitic samples have Nb/Ta-ratios in the range that would be expected for LP fluid related or magmatic rutile, respectively. In this respect, our dataset does not follow the discrimination previously proposed and applied to detrital rutile, which could indicate that some hydrothermal and pegmatitic rutile in detrital studies could be incorrectly classified as metamorphic.

### Significance of different defect types

Peak deconvolution has been performed on all measured spectra to identify different trace-element related H^+^-defects. In total, seven defects were identified amongst the investigated samples, with the spectra falling into four main classes, and some with subgroups. The most dominant H^+^-defects are related to Ti^3+^ and Fe^3+^, which have been observed in all investigated samples. The ratio of H^+^ related to Ti^3+^ over H^+^ related to Fe^3+^ is variable but shows that generally more H^+^ is coupled with Ti^3+^ than Fe^3+^, with a few exceptions. Seventeen metamorphic and one pegmatitic sample fall into the class only showing these two OH-bands. The most abundant spectral class was observed for 32 metamorphic and seven hydrothermal and pegmatitic samples. This class shows an Al^3+^-related H^+^-defect in addition to the Ti^3+^- and Fe^3+^-related defects. The relative amount of Al^3+^-related H^+^-defects is highly variable, although generally this defect appears to be important in natural rutile as it occurs in more than half of the investigated samples independently of metamorphic conditions and lithology, as well as in almost all hydrothermal and pegmatitic samples.

Hydrogen defects related to divalent cations are significantly less important overall, with the Fe^2+^-related defect being slightly more common than the Cr^2+^- and Mg^2+^-related defects. Metamorphic samples showing Fe^2+^- and/or Cr^2+^-related OH-bands can be associated to fluid-related veins or similar fluid-related alteration of the host rock. The presence of Fe^2+^ and Cr^2+^ in rutile indicates reducing conditions, as both Fe and Cr are redox sensitive elements. Thus, the abundance of H^+^-defects related to Fe^2+^- and/or Cr^2+^ might be a potential redox indicator. However, further investigations to evaluate this hypothesis are needed.

The shoulder on the Ti^3+^-related OH-band at ~ 3310 cm^−1^ identified in three of the hydrothermal and pegmatitic samples has not previously been described. For known OH-defect types, an approximate linear relationship is observed between the wavenumber of the OH-band and the ionic radius of the related trace element (Lueder et al. 2023). For trivalent cations, a smaller ionic radius relates to an OH-band at a higher wavenumber, whereas for divalent cations ionic radius and wavenumber of the OH-band are positively correlated, therefore divalent cations with larger ionic radii have related OH-bands at higher wavenumbers. Additionally, OH-bands related to divalent cations generally show a wider peak shape, due to a weaker OH-bond. The observed OH-band at ~ 3310 cm^−1^ is located at a wavenumber range between the OH-bands related to Fe^3+^ and Al^3+^, and has a sharp, narrow shape. Thus, it is most likely related to a trivalent cation. Following the linear relationship described by Lueder et al. (2023), it can be expected that a trivalent cation related to an OH-band at ~ 3310 cm^−1^ has an ionic radius of ~ 59–65 pm. Vanadium (V^3+^) with an ionic radius of 64 pm (Shannon 1976) is the only trace element that is abundant in rutile which falls into the range of ionic radii. Thus, the OH-band at ~ 3310 cm^−1^ is attributed to H^+^ coupled with V^3+^.

### H^+^ retention

As H^+^ potentially diffuses easily in mineral phases, including rutile (e.g., Caskey 1974; Johnson et al. 1975; Cathcart et al. 1979; Joachim-Mrosko et al. 2024), it is important to understand if the obtained H^+^ contents reflect the initial H^+^ contents and distributions in rutile during crystal growth, or if the H^+^ contents are partially to completely reset by diffusive H^+^ loss. However, H^+^ retention in natural rutile has not been comprehensively evaluated. FTIR mapping and H_2_O profiles can be used to assess the intra grain variability of H_2_O contents and thus the extent of H^+^ loss or H^+^ retention during metamorphism.

All three H_2_O maps show clear H^+^ zoning. The LT eclogite sample from Pfulwe, Western Alps, and the amphibolite facies sample from the Central Alps both show relatively symmetric zoning profiles (Fig. 5d, f). In the LT eclogite, H_2_O contents decrease from core to rim (Fig. 5c), whereas the amphibolite facies sample contains an increase towards the mantle and then a decrease towards the rim, with similar H_2_O contents at the rim and in the core in contact with an inclusion of white mica (Fig. 5a). The LT eclogite sample from Syros shows plateaus with constant H_2_O contents in both areas of high and low H_2_O contents and steep gradients between the different zones (Fig. 5e).

As all mapped samples show H_2_O zoning, H^+^ is at least partially retained and H_2_O contents are certainly not completely diffusively re-equilibrated. Particularly, plateaus of constant H_2_O content with step gradients between areas with high and low H_2_O contents indicate very limited diffusive resetting of H^+^. Thus, heterogeneous H_2_O contents are likely a primary, growth-related feature and are not purely diffusion related, i.e. did not experience extensive post-peak resetting. Whether the maximum measured H_2_O contents give original, unaltered H_2_O contents cannot be determined with absolute certainty, however, large (> 1000 µm) rutile grains likely preserve original (prograde to peak metamorphism) H_2_O contents in their core.

Most samples from HT lithologies (HT–HP eclogite facies, granulite facies) and UHP samples show rather large inter-grain variations in H_2_O contents (Fig. 4). In a Cr-rich HT–HP eclogite from the Central Alps (AA20-1) a clear relationship between H_2_O content and grain size is identified, with higher H_2_O contents in larger grains (Online Resource 3). This general trend can also be observed to a lesser degree in most samples from HT localities that show significant inter grain variability of H_2_O contents. Further, nominally dry samples from UHP and granulite facies conditions generally have very small grain sizes of ~ 10–75 μm.

**Fig. 7:**
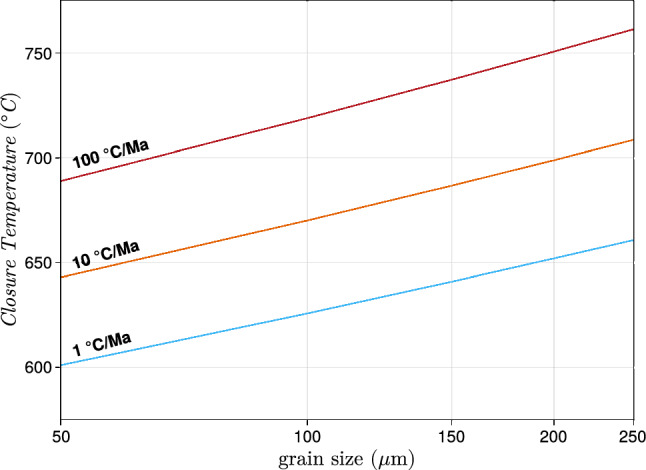
Closure temperatures after Dodson (1973) for coupled H^+^ diffusion and oxygen vacancies (diffusion data for oxygen vacancies of Moore et al. 1998). depending on the grain size of rutile for cooling rates of 1 °C/Ma (blue line), 10 °C/Ma (orange line) and 100 °C/Ma (red line)

Hydrogen diffusion in rutile has been studied experimentally in nominally pure rutile under oxidizing conditions (e.g., Caskey 1974; Johnson et al. 1975; Cathcart et al. 1979), as well as natural rutile (Joachim-Mrosko et al. 2024). Hydrogen associated with trivalent Ti could be lost by diffusion with a concomitant oxidation of Ti (e.g., Ti^3+^ + H^+^ → Ti^4+^). This reaction does not involve diffusion of any metal cation and is potentially very fast. Using H diffusion data of Johnson et al. (1975) and using the formulation of closure temperature after Dodson (1973), closure temperatures for H^+^ diffusion in rutile could be as low as ~ 200–240 K (< 0 °C) for cooling rates of 1–100 °C/Ma and grain sizes of 50–250 μm. This would imply active diffusive H^+^ loss out of rutile at ambient conditions, which can be excluded due to observable H^+^ zoning seen in this study. Therefore, in natural, non-pure rutile, the coupling of H^+^ with di- and trivalent cations influences the ability of H^+^ to diffuse out of the rutile crystal. The loss of H^+^ out of rutile would lead to a charge deficit, that must be balanced by the formation of oxygen vacancies or the coupled diffusion of other cations with H^+^. Charge balanced can be achieved via: (i) The exchange of a pentavalent cation for a tetravalent cation (e.g., Fe^3+^ + Ti^4+^ + H^+^ → Fe^3+^ + Nb^5+^), for which diffusion of pentavalent cations into rutile would be controlling H^+^ diffusion. According to diffusion data from Dohmen et al. (2019), closure temperatures for Nb in rutile for grain sizes of 50–250 μm and cooling rates of 10 °C/Ma are in a range of 590–660 °C. Diffusive loss of tetravalent cations (e.g. Zr) have closure temperatures in a similar range of ~ 570–730 °C, for grain sizes of 50–250 μm and cooling rates of 10 °C/Ma (Cherniak et al. 2007; Kohn et al. 2016; Dohmen et al. 2019). However, Ti diffusion in rutile is orders of magnitude faster (e.g. Hoshino et al. 1985), making diffusion of other tetravalent cations less significant. Additionally, it can be assumed that transport of HFSE, such as Nb and Ta, in the rock matrix and therefore, availability of pentavalent cations, could be a rate-limiting factor. Thus, this charge balance mechanism is likely of lesser importance.

(ii) The exchange of a trivalent cation for a tetravalent cation (e.g., Fe^3+^ + H^+^ → Ti^4+^). In this case, diffusion rates of trivalent cations are rate limiting. Joachim-Mrosko et al. (2024) show correlated diffusion profiles for H^+^, Al, and Fe and report diffusion data for Al, with diffusion coefficients several orders of magnitude slower than reported values for H^+^ (e.g. Johnson et al. 1975). At temperatures of ~ 600–650 °C (closure temperature for Nb in rutile, see above), diffusion of Al in rutile is 3–4 orders of magnitude slower, which then relates to even higher closure temperatures. As Fe shows very similar diffusion profiles to Al (Joachim-Mrosko et al. 2024), it might be expected that Fe diffusion in natural rutile occurs at similar rates as Al diffusion, and thus much slower rates than previously reported (e.g., Sasaki et al. 1985). Therefore, Al diffusion rates in rutile are assumed to be representative for other relevant trivalent cations.

(iii) The formation of oxygen vacancies to charge balance trivalent cations (e.g., 2H^+^ + Fe_2_O_4_ → Fe_2_O_3_). Oxygen vacancies have been shown to be important for charge balance in rutile (e.g., Bromiley et al. 2004; Lueder et al. 2023; Joachim-Mrosko et al. 2024). Oxygen diffusion in rutile is 2–3 orders of magnitude faster than Al diffusion and ~ 1 order of magnitude slower than Nb diffusion (Moore et al. 1998). Closure temperature for grain sizes of 50–250 μm and cooling rates of 10 °C/Ma are in a range of 640–710 °C (Fig. 7).

As charge balance mechanisms involving pentavalent cations are unlikely due to limited availability of pentavalent cations from the rock matrix, charge balance mechanisms (ii) and (iii), only involving trivalent cations and oxygen vacancies, are the likely dominating factors. Oxygen diffusion in rutile has a closure temperature of ~ 600–750 °C for typical grain sizes and cooling rates (Fig. 7). Trivalent cations (e.g., Al, Joachim-Mrosko et al. 2024) have slower diffusion coefficients at equivalent temperatures, therefore even higher closure temperatures. Additionally, formation of oxygen vacancies is not dependent on the availability of cations within the rock matrix and therefore the most relevant charge balance mechanism which determies closure temperatures of H^+^ diffusion in rutile.

Based on the previous discussion, rutile can most likely preserve its formation/growth H^+^ and trace element signatures up to peak metamorphic temperatures of ~ 600–650 °C. This would result in the partial or total resetting of H_2_O contents in rutile with higher peak metamorphic temperatures, and that large grains are more likely to preserve the initial H_2_O contents in their core. The vein-related sample from an eclogite from the Central Alps with peak metamorphic conditions of 2.5 GPa and 750 °C and grain sizes of up to ~ 2 mm may represent this case, as expected closure temperatures for oxygen diffusion in rutile are ~ 870 °C (diffusion data from Moore et al. 1998). Thus, large grains formed at HT peak metamorphic conditions can preserve original H_2_O contents, at least in their core, whereas it can be expected that H_2_O contents decrease significantly towards the rim.

Consequently, H_2_O zoning in rutile with peak metamorphic temperatures below 600 °C, as typical for cold subduction samples, are interpreted as a primary feature. Importantly, this indicates that heterogeneous H_2_O contents can develop during rutile growth.

### Rutile H^+^- and trace-element chemistry as indicator for cold subduction conditions

Rutile trace-element chemistry is frequently used in provenance analyses to infer age, composition and formation temperature of the protolith (e.g. Zack et al. 2004a; Tomkins et al. 2007; Meinhold et al. 2008; Meinhold 2010; Zack and Kooijman 2017; Agangi et al. 2019; Kohn 2020; Pereira et al. 2021). Estimating formation pressures of rutile is significantly more difficult. Rutile can potentially have inclusions of pressure-sensitive phases such as coesite (e.g., Hart et al. 2016), but these are rare occurrences. Dark brown colors of rutile complicate the identification of mineral inclusions, and inclusion-barometry has not yet been tested and calibrated. Thus, it is necessary to identify an alternative method of inferring LT–HP or UHP conditions of detrital rutile, as indicative of ‘cold’ subduction.

**Fig. 8:**
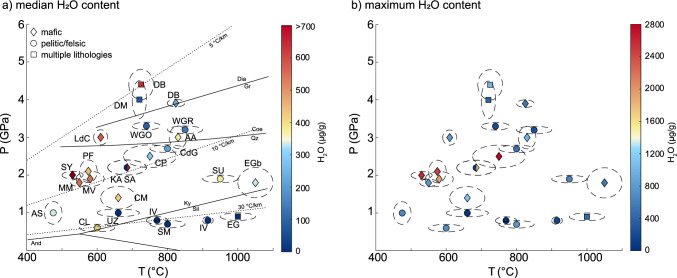
Median (**a**) and maximum (**b**) H_2_O contents of all analysed samples for each locality, depending on peak P–T-conditions. Diamonds indicate sample localities with mafic lithologies, circles indicate localities with felsic lithologies, and squares indicate sample localities with both mafic and felsic lithologies. The symbol color indicates the median H_2_O content of all samples that have been analysed for a single locality. *AA* Alpe Arami, *AS* Alpe Senevedo Superiore, *CM* Val Cama, *CL* Campolungo, *CP* Alpe Capoli, *CdG* Cima di Gagnone, *DB* Dabie Shan, *DM* Dora-Maira, *EG* Eastern Ghats, *EGb* Erzgebirge, *IV* Ivrea, *KA* Koralpe, *LdC* Lago di Cignana, *MM* Monte Mucrone, *PF* Pfulwe, *SA* Saualpe, *SM* Serre Massif, *SU* Sudetes, *SY* Syros, *UZ* Ulten Zone, *WGR* Western Gneiss Region, *WGO* West Gondwana Orogen. P–T conditions are from the literature with reported uncertainties as elipses, for references see Table 1

We see a clear relationship between median and maximum H_2_O content of rutile and peak metamorphic conditions of the host rock (Fig. 8). In addition to the median H_2_O contents, we also consider the maximum, as the maximum is closest to the maximum H_2_O content obtained during prograde rutile growth in the case of diffusive H^+^ loss from the rutile crystals. The correlation between H_2_O content in rutile and peak conditions experienced by the metamorphic samples is compelling. Two general trends are observed for H_2_O contents of rutile: (i) Rutile from localities with higher peak metamorphic pressures show higher H_2_O contents compared to samples from lower pressure localities. This is seen for LT eclogite facies samples that formed at ~ 500–600 °C and pressures of 1.2–2.1 GPa and have median H_2_O contents > 450 μg/g and maximum H_2_O contents above ~ 800 μg/g, in comparison to amphibolite facies samples which formed in a similar temperature range but at lower pressures of 0.8–1.0 GPa with maximum H_2_O contents below ~ 500 μg/g. Similarly, LP granulite facies samples are on average nominally dry, with maximum H_2_O contents below ~ 100 μg/g, whereas HP granulite facies samples have on average ~ 350 μg/g H_2_O. (ii) Rutile from localities with peak metamorphic temperatures above 600–700 °C show lower H_2_O contents than samples with similar peak metamorphic pressures but lower temperatures. This is seen when comparing LT eclogite facies samples which have a median H_2_O content of > 450 μg/g and maximum H_2_O contents of ~ 800–2500 μg/g, with HP granulite facies and HT–HP eclogite facies samples that formed at similar pressures and have average H_2_O contents of ~ 300–350 μg/g and lower maximum H_2_O contents (~ 250–1000 μg/g). A likely explanation for this is a partial loss of H^+^ by diffusion as discussed above, which is supported by the unusually high maximum H_2_O contents in one sample from a rutile vein in an eclogite from the Central Alps with large grain sizes (> 1000 μm). Both trends together lead to very distinctive, high H_2_O contents in ‘cold’ subduction related LT eclogite facies rutile, with median H_2_O contents of ~ 450–2000 μg/g (Fig. 8a) and maximum H_2_O contents of ~ 800–2500 μg/g (Fig. 8b).

The studied LT eclogite facies samples with high H_2_O contents are mafic and thus high in bulk Fe and Al, which translates to high Fe contents in rutile from these samples, far exceeding the H_2_O content. Thus, in this instance, the incorporation of H^+^ into rutile should not be limited by the presence of trivalent cations for coupled substitution, but rather by H_2_O fugacity. As f(H_2_O) increases with increasing pressures, samples with relatively higher peak metamorphic pressures have higher H_2_O contents in rutile. Consequently, H_2_O in rutile is proposed as a suitable pressure indicator for ‘cold’ subduction related mafic rocks. Thus, rutile H_2_O/Zr ratios in mafic samples can indicate the thermal gradients, with Zr as temperature proxy (Zack et al. 2004a; Tomkins et al. 2007; Kohn 2020) and H_2_O as pressure indicator. However, maximum observed H_2_O can only record minimum pressures, as retrograde/decompressional H^+^ loss is prevalent in rutile from HT samples. This leads to a preservation bias for HT-HP rutile, which are likely to record the retrograde path.

High H_2_O/Zr-ratios are equivalent to low geothermal gradients (i.e., high P/T) and low H_2_O/Zr-ratios to high geothermal gradients (i.e., low P/T). This applies only to rutile with pristine H_2_O contents that are unaffected by diffusive H^+^ loss. Rutile grains that have partially lost their H^+^ develop lower H_2_O/Zr-ratios, thus overlapping with ‘warm’ and ‘hot’ thermal gradients. This is seen for samples from Saualpe and UHP samples from Dora Maira, Dabie Shan, and Mali, suggesting a potential diffusive H^+^ loss at high temperatures. Hydrothermal and pegmatitic rutile generally have high H_2_O contents and low Zr contents, leading to high H_2_O/Zr-ratios.

**Fig. 9:**
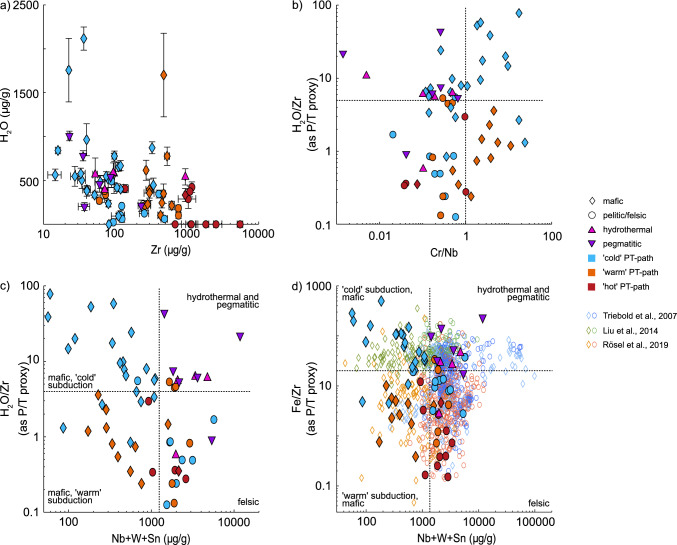
**a** H_2_O–Zr diagram: Zr relates to formation temperature of metamorphic rutile (e.g. Zack et al. 2004a), H_2_O is used as proxy for formation pressure. **b** H_2_O/Zr-ratio vs. Cr/Nb. **c** H_2_O/Zr-ratio (i.e. P/T) as proxy for thermal gradient versus Nb + W + Sn as proxy for lithology. The field for ‘cold’ subduction lies at a H_2_O/Zr ratio of 4 and a total Nb + W + Sn-content of 1250 μg/g. **d** Fe/Zr-ratio vs. Nb + W + Sn, with detrital data from Triebold et al. (2007), Liu et al. (2014), and Rösel et al. (2019). Mafic samples are shown as diamonds, felsic samples as circles. Blue symbols indicate ‘cold’ subduction thermal gradients (~ 150–350 °C/GPa), orange ‘warm’ thermal gradients (~ 275–575 °C/GPa) and red ‘hot’ thermal gradients (~ 500–1500 °C/GPa). Hydrothermal and pegmatitic samples are depicted as pink and purple triangles respectively. Symbols represent median values for each sample with uncertainties in **a** as median absolute deviations

When plotting H_2_O contents in rutile as pressure indicator vs. Zr in rutile as temperature proxy (Fig. 9a), there is a significant overlap of LT, ‘cold’ subduction mafic and felsic samples with samples from ‘warm’ and ‘hot’ geotherms, LT metapelitic samples, as well as hydrotheral, and pegmatitic samples. Thus, it is necessary to differentiate between LT eclogite facies mafic rutile, from amphibolite facies (metapelitic), hydrothermal, and pegmatitic rutile. As rutile trace-element geochemistry is strongly dependent on host-rock composition, discrimination diagrams can be a valuable tool to infer the source protolith. Differentiating mafic and felsic rutile based on Nb and Cr contents (Zack et al. 2004a) is reliable, as confirmed by our dataset. However, the overlap of hydrothermal and pegmatitic rutile with felsic rutile may lead to an inaccurate evaluation of detrital grains. It is thus vital to differentiate pegmatitic and hydrothermally derived or altered rutile (which are not related to subduction) from other felsic rutile in a detrital data set. High W and Sn contents are often used as a tool to distinguish hydrothermal and magmatic rutile from metamorphic rutile (e.g., Clark and Williams-Jones 2004; Meinhold 2010; Agangi et al. 2019; Pereira et al. 2021). However, our dataset shows a large overlap in W and Sn contents for allfelsic samples. Thus, W and Sn contents are not sufficient for the differentiation of mafic and felsic rutile. Felsic metamorphic, hydrothermal and pelitic rutile have high Nb contents resulting in low Cr/Nb ratios. However, as mafic rutile with < 800 μg/g Nb can potentially also have low Cr/Nb ratios and low H_2_O/Zr-ratios due to H^+^-loss, there is still a significant overlap of the different lithologies, meaning that Cr/Nb ratios are not a viable proxy for lithologies (Fig. 9b).

Combining Nb-, W-, and Sn contents as constraints on host-rock type with H_2_O/Zr ratio as proxy for thermal gradients allows a clearer differentiation of rutile from different lithologies (Fig. 9c). In our dataset four groups can be distinguished (Fig. 9c); (i) most hydrothermal and pegmatitic rutile at high H_2_O/Zr ratios and high Nb + W + Sn; (ii) most felsic rutile, independent of subduction thermal gradient, at low H_2_O/Zr ratios and high Nb + W + Sn; (iii) ‘warm’ subduction, mafic rutile at low H_2_O/Zr ratios and low Nb + W + Sn; and iv) mostly mafic, ‘cold’ subduction rutile, at high H_2_O/Zr ratios and low Nb + W + Sn. Thus, H_2_O/Zr ratios above 4 together with total Nb + W + Sn contents below 1250 μg/g in rutile are the best indicators of cold subduction conditions in mafic rocks (e.g. igneous oceanic crust).

Additionally, Fe and H_2_O contents are correlated, low Fe and H_2_O is seen in granulite facies samples, intermediate contents in ‘warm’ subduction samples, and high Fe and H_2_O in ‘cold’ subduction, hydrothermal and pegmatitic samples (Fig. 2h). Thus, high Fe contents, which can be readily measured with EPMA (although limited by secondary fluorescence brought on by neighboring Fe-rich phases, e.g. garnet or ilmenite) or LA–ICP–MS, may be used as a first order approximation for high H_2_O contents. Hydrogen in rutile is coupled with different di- and trivalent cations, however, Ti^3+^ and Fe^3+^ are the most dominant H^+^ related defects (see above). High Fe contents in rutile can thus directly account for higher H_2_O contents. We propose that, in a first instance, the Fe/Zr ratio may be used as alternative for H_2_O/Zr ratio (Fig. 9d) with the caveat that Fe contents strongly depend on lithology, as rutile from felsic samples show generally lower Fe contents than from mafic samples. Figure 8d shows a comparison of our data for Fe/Zr ratios vs. total Nb + W + Sn contents and literature data for detrital rutile from three different localities for which felsic and mafic rutile are differentiated after the Cr-Nb-discrimination diagram (Meinhold et al. 2008). Late Ordovician detrital rutile from Saxo-Thuringia (Rösel et al. 2019) shows high Nb + W + Sn contents of > 800 μg/g and generally low Fe/Zr contents < 20 for felsic rutile. Mafic rutile from this study have highly variable Nb + W + Sn contents and mostly low Fe/Zr-ratios, falling into the field for ‘warm’ subduction, with few grains showing high Fe/Zr ratios (~ 20–200) indicative of ‘cold’ subduction related mafic rutile. Felsic detrital rutile from the Erzgebirge (Triebold et al. 2007) have high Nb + W + Sn contents and mostly high Fe/Zr-ratios, thus mostly falling into the fields for hydrothermal and pegmatitic and felsic rutile. Mafic rutile from the study of Triebold et al. (2007) has highly variable Fe/Zr-ratios and Nb + W + Sn contents, falling into the fields for hydrothermal and pegmatitic, felsic, and ‘warm’ subduction mafic rutile, defined according to the results of our study. As both source areas represent mainly lithologies from ‘warm’ and ‘hot’ geothermal gradients, the observed results align well with expectations from our own data set. Felsic detrital rutile from Dabie Shan (Liu et al. 2014) have high Nb + W + Sn contents and Fe/Zr-ratios > 20. Thus, all felsic rutile falls into the field of hydrothermal and pegmatitic rutile defined in our study. As the classification of detrital rutile after Cr-Nb-contents (Meinhold et al. 2008) does not differentiate between felsic, hydrothermal, and pegmatitic rutile, it is reasonable that rutile classified as ‘felsic’ (i.e., derived from a felsic host rock) can be hydrothermal and/or pegmatitic in origin. Mafic rutile from LT to UHP eclogite facies from Dabie Shan (Liu et al. 2014) has mostly low Nb + W + Sn contents < 2000 μg/g and high Fe/Zr-ratios > 20, thus falling into the field for ‘cold’ subduction mafic rutile, as would be expected. Consequently, Fe/Zr-ratios together with total Nb, W, and Sn contents should only be applicable to mafic LT eclogite facies rutile, as an approximation for H_2_O/Zr-ratios for the identification of ‘cold’ subduction.

Consequently, we propose the use of H_2_O/Zr vs. total Nb + W + Sn discrimination diagrams for the identification of ‘cold’ subduction related rutile in the detrital record. This, however, should only be applied for the identification of mafic ‘cold’ subduction related rutile, and not for the positive identification of rutile from relatively warmer subduction geothermal gradients, rutile from metamorphic rocks with felsic bulk rock composition, hydrothermal, or pegmatitic rutile. Additionally, not all mafic ‘cold’ subduction related rutile will fall into the related area of the discrimination diagram. Diffusive H^+^-loss will lead to lower H_2_O/Zr ratios, thus falling below the threshold for ‘cold’ subduction. Mafic rutile with high Nb and Cr contents can have total Nb + W + Sn contents above the threshhold of 1250 μg/g. Thus, not all mafic ‘cold’ subduction related rutile will be identified using this method. However, the strength of this method is the unlikely false positive classification of non- ‘cold’ subduction rutile as being ‘cold’ subduction related. Rutile from HT-LP peak metamorphic conditions, i.e., related to ‘hot’ geothermal gradients, has initially high Zr and low H_2_O contents. During cooling, Zr and H_2_O can diffusively re-equilibrate down to temperatures of ~ 600–700 °C and 600–650 °C for typical grain sizes and cooling rates, respectively. This would lead to low Zr contents, similar to LT-HP rutile. However, as H_2_O contents are also significantly lower in the rutile grains, H_2_O/Zr ratios will still remain below the threashhold for ‘cold’ subduction. Rutile from HT-HP peak metamorphic conditions has initial high Zr and high H_2_O. Decompression will lead to H^+^-loss, due to the pressure dependence of H_2_O in rutile, ultimately reaching similar Zr and H_2_O contents as rutile from HT-LP peak metamorphic conditions. Therefore, rutile from neither of these resetting paths will be positively identified as related to ‘cold’ dubduction in the proposed discrimination diagram. Isobaric cooling of rutile from HT-HP peak metamorphic conditions will result in diffusive loss of Zr and H_2_O down to their respective closure temperatures. As the closure temperatures for Zr are higher than the expected closure temperatures for H^+^ in rutile, at the same grain size and cooling rate, Zr contents will reflect higher temperature conditions. Therefore, Zr contents in isobarically cooled HT-HP peak metamorphic rutile will be higher compared to rutile from HP-LT peak metamorphic conditions. Thus, H_2_O/Zr ratios of cooled HT-HP will be lower, falling below the threshold for ‘cold’ subduction. It is therefore unlikely that metamorphic rutile unrelated to ‘cold’ subduction would be misidentified as being related to ‘cold’ subduction.

## Conclusions

We studied H_2_O and trace-element contents in rutile from various lithologies and P–T conditions to evaluate rutile as a potential indicator of cold thermal gradient subduction conditions. H_2_O contents in rutile vary widely, from nominally dry granulite facies rutile to > 1000 μg/g in LT eclogite facies rutile, making rutile one of the most water-rich nominally anhydrous minerals. Rutile from LT–HP peak metamorphic conditions has significantly higher H_2_O than other metamorphic rutile, with only hydrothermal- and pegmatitic rutile having H_2_O contents comparable to LT eclogite facies rutile. Evidence from high-resolution mapping suggests that H_2_O contents are generally preserved in the low to mid temperature samples and H_2_O incorporation into rutile can be heterogeneous due to depletion of H_2_O in the rock during prograde rutile growth. H_2_O/Zr ratios are a proxy for thermal gradients of metamorphic rutile (i.e. P/T), whereas high Nb, W, and Sn contents identify felsic and hydrothermal- or pegmatitic rutile. High H_2_O/Zr -ratios (> 4) together with low Nb + W + Sn contents (< 1250 μg/g) are indicative of ‘cold’ subduction related rutile from mafic rocks. In the absence of H_2_O analyses in rutile, Fe/Zr-ratios might be used as a first order approximation for H_2_O/Zr-ratios to identify mafic LT eclogite facies rutile. Felsic rutile should not be evaluated using Fe/Zr ratios due to a strong dependence of Fe contents on lithology. The developed systematics can be used in the search of ‘cold’ subduction conditions in early Earth based on detrital rutile.

### Supplementary Information

Below is the link to the electronic supplementary material.

Supplementary file1 (PDF 762 KB)

Supplementary file2 (XLSX 228 KB)

Supplementary file3 (XLSX 199 KB)

## Data Availability

Raw data form FTIR and LA-ICP-MS measurements can be made available upon request.
