# Supplementary material for: H2O-rich rutile as an indicator for modern-style cold subduction
Source: Contrib Mineral Petrol. 2024 Mar 10;179(3):26. doi: 10.1007/s00410-024-02107-2 (PMC10955008; doi:10.1007/s00410-024-02107-2)
Supplement: Supplementary file 1 — Supplementary file1 (PDF 762 KB) [file 410_2024_2107_MOESM1_ESM.pdf]

Sample descriptions

Amphibolite Facies Samples

The three samples from Alpe Senevedo Superiore, Val Malenco, Italian Alps (AS19-1, AS19-2, AS19-3) record amphibolite facies conditions of 1.0 ± 0.3 GPa and 475 ± 25 °C (Bissig and Hermann, 1999). All are metapelitic with different mineral assemblages. Sample AS19-1 contains sub-rounded hexagonal garnet grains of 0.5–2 mm size, µm–cm sized phengitic white mica, and quartz within the matrix and as quartz-layer. Rutile occurs as ~200 µm–sized grains within the matrix and as large cm–sized grains, with ilmenite exsolution needles associated with the quartz-layer. Samples AS19-2 and AS19-3 contain garnet and white mica as well as chlorite and amphibole, with a larger grain size of 200–500 µm and a strong preferred orientation in sample AS19-2, and a significantly smaller grain size (< 150 µm) and un-oriented grains in AS19-3. In AS19-2 rutile is fine grained and homogeneously distributed throughout the sample, while in AS19-3 rutile occurs both within the matrix and as larger grains associated with quartz lenses, similar to sample AS19-1.

Sample 19-JH-03 comes from a metapelite from Campolungo, Central Alps, which contains quartz, white mica, biotite, plagioclase, garnet, minor allanite, and zircon. Rutile has a grain size 20–150 µm. Peak metamorphic conditions are 0.6 ± 0.1 GPa and 600 ± 50 °C (Boston et al., 2017).

Two samples from Val Cama, Central Alps, record peak metamorphic conditions of 1.2 ± 0.2 GPa and 690 ± 40 °C (Dale and Holland, 2003). Sample CM20-2 displays a mineral assemblage of amphibole, garnet with pronounced amphibole rims, plagioclase, minor spinel, magnetite, titanite, rutile, and accessory zircon. Rutile within the matrix has grain sizes of up to 1 mm and often occurs with ilmenite intergrowth. Additionally, rutile occurs within a vein, associated with clinozoisite and grain sizes of up to 5 mm. Vein related rutile grains show intergrowth with ilmenite and titanite rims. Sample CM20-6 is a metarodingite containing garnet, clinopyroxene, often with amphibole rims, minor epidote, titanite, ilmenite and accessory zircon. Rutile occurs as vein intergrown with titanite and ilmenite and grain sizes of 1–4 mm.

One sample from a metapelite from the Ulten Zone, Northern Italy (Ulten) records peak metamorphic conditions of 1.0 ± 0.1 GPa and 660 ± 60 °C (Braga et al., 2007). The sample has a mineral assemblage of mm-sized garnet and kyanite prophyroblasts in a lineated matrix of plagioclase and quartz, with inter layered biotite. Rutile occurs as aggregates of multiple < 70 µm sized grains.

Eclogite Facies Samples

LT Eclogite Facies Samples

Five rutile single grain samples from Monte Mucrone, Sesia Zone, Italian Western Alps (M-E1, M-E2, M-Q1, M-Q2, M-Q3), which experienced peak metamorphic conditions of 1.8 ± 0.1 GPa and 550 ± 50 °C (Vho et al., 2020) were analysed. All grains come from eclogite lenses within micaschists and paragneisses with a mineral assemblage of

omphacite, garnet, glaucophane, paragonite, and phengitic white mica. The selected rutile grains have a size of 1–5 mm with pronounced ilmenite exsolution needles.

One sample from Syros, Cyclades, Greece (SY21-31) is from a metabasic blueschist-eclogite mélange. It was taken as rutile single grain from the Kampos subunit, which is comprised of an omphacite-phengite matrix with garnet and glaucophane phenocrysts and minor quartz, albite, calcite, epidote and rutile (Laurent et al., 2018). The rutile grain has small ilmenite exsolution needles. Peak metamorphic conditions are approximately 2.0 ± 0.1 GPa and 530 ± 20 °C. Two samples from Syros (SY-KM1, SY-KM2) come from the northern part of the island, the precise sample locality is unknown. Sample SY-KM1 belongs to a placer deposit sourcing eclogite facies rutile, with the precise sample locality unknown and sample SY-KM2 is a single ~1x1x1 cm sized rutile grain from an eclogite unit. Peak metamorphic conditions are comparable to sample SY21-31.

Three samples from a large eclogite body from Monviso, Western Alps contain omphacite, garnet, quartz, amphibole and minor phengite. Samples MVE4 and MVE12 have minor rutile within the matrix, while rutile in sample MVE2 forms cluster of several 30–300 µm–sized grains that are oriented along the foliation, forming lenses of up to 1 cm length. Peak metamorphic conditions are 1.9 ± 0.2 GPa and 580 ± 40 °C (Schwartz et al, 2000).

The six samples from Pfulwe, Zermatt-Saas-zone, Western Alps, are part of an ophiolite sequence that has been metamorphosed at peak conditions of 2.1 ± 0.3 GPa and 575 ± 25 °C (Barnicoat and Fry, 1986). Sample PF18-14 comes from an Fe-Ti-gabbro, containing partially resorbed, up to cm–sized, inclusion-rich garnet with retrograde amphibole rims in a matrix of chloritoid, omphacite, glaucophane relicts, and paragonite. Rutile occurs mainly as aggregate of several 100 grains of 100–800 µm size that is interpreted to be a pseudomorph replacing an ilmenite layer. Larger grains show dark blue tinted cores and some have ilmenite exsolution needles. Sample PF18-26b also comes from a metagabbro. Garnet, glaucophane and omphacite have grain sizes of up to 1 cm. The matrix contains smaller omphacite, paragonite, fibrous quartz (chalcedony), epidote and 50–250 µm–sized rutile. Samples PF18-25c and PF18TB01 were taken from the rims of large pillow basalts. The metamorphic assemblage consists of garnet, omphacite, epidote, paragonite and phengitic white mica, minor quartz, chlorite and glaucophane. Small ~200 µm–sized rutile is present in association with omphacite- and mica-rich zones. Sample PF21-01 comes from a blueschist, containing cm–sized, euhedral garnet in a matrix of glaucophane, phengitic white mica, quartz and 50–150 µm–sized rutile. Sample PF21-02 is a ~2 cm long single rutile grain taken from a quartz vein within the metagabbro, with a dark brown color.

Sample C31 sample from Lago di Cignana, Italian Western Alps, formed at peak metamorphic conditions of 3.0 ± 0.2 GPa and 610 ± 20 °C (Reinecke, 1998; Groppo, 2009). The mineral assemblage comprises strongly zoned porphyroblastic garnets with quartz, omphacite, and amphibole inclusions, in a matrix of omphacite, glaucophane, and phengitic white mica. Garnet and glaucophane are partially replaced by Ca-rich amphibole. Rutile occurs as 50–300 µm–sized grains throughout the matrix and as < 50 µm–sized inclusions in garnet and glaucophane.

One metapelite samples from Koralpe, Eastern Alps and two eclogite samples from the Saualpe eclogite type locality, Eastern Alps were studied. Peak metamorphic conditions are 2.2 ± 0.2 GPa and 685 ± 55 °C (Thöni et al., 2008). The metapelite sample from Koralpe contains rounded 0.5–7 mm sized garnets in a matrix of quartz, plagioclase and white mica. Garnet and matrix minerals show retrograde alteration to amphibole and biotite. Rutile is present throughout the

matrix and has a grain size of 30–200 μm. Sample Sau3 has a mineral assemblage of garnet, omphacite, quartz and phengitic white mica, with a homogeneous texture and grain sizes of 0.3–2 mm. Sample Sau2 contains garnet, omphacite, and quartz, with kyanite and amphibole. The grain size is significantly larger compared to sample Sau3.

High-T – High-P Eclogite Facies Samples

Four eclogite samples from Alpe Capoli, Central Alps, (CP16-03B, CP16-04, CP18-03A, CP18-03B) record peak metamorphic conditions of 2.5 ± 0.3 GPa and 750 ± 75 °C (Dale and Holland, 2003). Samples CP16-04, CP18-03A, CP18-03B have mineral assemblages containing garnet, omphacite, quartz, and minor phengitic white mica. All three samples have an extensive amphibolite facies overprint comprised of amphibole rimes and symplectites around garnet and omphacite. Rutile grains have a size of 50–100 μm and a light brown color. Sample CP16-03B is part of a rutile vein within the eclogite and contains only < 2 Vol% inclusions of omphacite, amphibole and white mica. The rutile grains have a size of 0.5–2 cm and often show ilmenite exsolution lamella and dark, blue tinted cores.

Sample CdG19/36 is a metapelite from Cima di Gagnone, Central Alps. It contains cm-sized, often atoll shaped garnet, white mica, quartz, kyanite, minor omphacite, zircon, and epidote with retrograde plagioclase and biotite. Peak metamorphic conditions were 2.7 ± 0.1 GPa and 800 ± 50 °C (Piccoli et al., 2021).

Four samples from the eclogite body of Alpe Arami, Central Alps, were analyzed. All samples have peak metamorphic conditions of 3.0 ± 0.3 GPa and 830 ± 25 °C (Trommsdorff et al., 2000). Sample AA16-11 and AA21-02 contain elongated, 0.5–2 mm sized garnet and omphacite grains. Omphacite is rimed by retrograde amphibole and fractures are filled with small quartz grains. Sample AA21-02 show additional symplectite formation. Rutile occurs as mm-sized grains and is elongated parallel to the orientation of omphacite and garnet in sample AA16-11. Sample AA20-1 has a mineral assemblage of garnet, omphacite and kyanite. Garnet can occur in grain sizes up to 3 cm and is strongly retrogressed in contact with omphacite. Smaller garnet grains can be found throughout the omphacite domain, mostly rimed with amphibole. The assemblage is cut by a large kyanite-quartz vein. Rutile occurs in association with garnet as 200–500 μm sized, fractured grains. Sample AA21-01 is a garnet-pyroxenite, containing mainly garnet, with less omphacite and partial alteration to amphibole. Rutile is homogeneously distributed throughout the sample and has grain sizes of 30–500 μm.

UHP Samples

Two samples from different lithologies within the West Gondwana Orogen, Mali were studied. Peak metamorphic pressures of all three samples are 3.3 ± 0.1 GPa and 740 ± 40 °C (Ganade de Araujo et al., 2023). Both samples S-508 and S520 were taken from a quartzite that is interlayered within mafic eclogites.

Two metapelites from Fjortoft, Western Gneiss Region, Norway (WG17-01, FJ-RT) come from the UHP domain with peak metamorphic conditions of 3.2 ± 0.1 GPa and 850 ± 50 °C (Butler et al., 2015; Hacker et al., 2015; March et al., 2022). Both samples contain large, mm-sized, inclusion-rich garnets in a matrix of quartz, kyanite, and feldspars, with minor white mica, Retrograde biotite is abundant in association with garnet and forms bands around most garnet grains. Rutile occurs at grain sizes < 75 μm mainly as inclusions in garnet, less within the matrix.

Six samples of different lithologies from the Dora-Maira Massif, Western Alps, record UHP metamorphic conditions of 4.0 ± 0.5 GPa and 720 ± 20 °C (Hermann, 2003; Gauthiez-Putallaz et al., 2016). Sample DM1c comes from a

whiteschist and contains up to cm-sized garnets in a foliated matrix of quartz (retrograde from coesite; Chopin, 1984), kyanite and phengite. Garnets are rimed by retrograde kyanite, talc and phlogopite. Rutile occurs mainly as inclusions in garnet with grain sizes < 50 µm. Sample DM2a comes from a vein of slightly different composition within the whiteschist. It contains a mainly quartz and retrograde talc, with smaller (~1 mm), rounded garnet grains and kyanite. Fine grained rutile (< 50 µm) occurs throughout the matrix and as inclusions in garnet. Samples DM5 and DM10 come from metapelites with a mineral assemblage of garnet, phengite, quartz and kyanite. Garnet, phengite and kyanite have larger grain sizes up to cm-scale in DM5, while sample DM10 has significantly smaller grains (<3 00 µm). Further, retrograde amphibole is abundant in sample DM5, while sample DM10 contains mainly white mica as alteration product. Sample DM30 is a strongly foliated, fine grained (20–100 µm) eclogite, containing garnet, omphacite, phengite, quartz, and rutile. Sample DM9 was taken from the contact between phengite-bearing eclogite and metapelite. It contains both the fine grained eclogite consisting of garnet, omphacite, quartz, phengite and rutile, as well as a metapelite with cm-sized phengite and a fine grained (100–200 µm) matrix of quartz, garnet, kyanite and retrograde amphibole. Rutile within the metapelite has grain sizes up to 0.5 mm and ilmenite exsolution needles.

Five samples from the Dabie Orogen, central China, were analyzed, including three carbonate-bearing eclogite samples from the Shuanghe area, Central Dabie (DB1, DB2, SH02-3) and two samples of mafic lenses within marble-paragneisses from the Ganjialing area, Central Dabie (DB6, DB7). The three eclogite samples from Shuanghe experienced peak metamorphic conditions of 3.9 ± 0.1 GPa and 825 ± 25 °C (Liu et al., 2020). They contain garnet, quartz, omphacite, and retrograde amphibole. DB1 has a massive texture with large (5–7 mm) garnets and thin amphibole rims around omphacite. Rutile has grain sizes of up to ~700 µm. Sample DB2 is layered, with one layer containing fine grained quartz and garnet, with rutile homogeneously throughout (< 50 µm). The second layer contains garnet, quartz, partially as veins, and omphacite with extensive amphibole rims, as well as minor biotite. Sample SH02-3 has a smaller grain size compared to DB1 and omphacite is more extensively replaced by amphibole. Rutile is present thorough the matrix and has grain sizes of 100–200 µm. The assemblage is cut by a coarse grained garnet-bearing quartz vein that contains large (~500 µm) rutile grains with ilmenite needles. The two samples from Ganjialing reached peak metamorphic conditions of 4.4 ± 0.4 GPa and 725 ± 35 °C (Liu et al., 2014). Sample DB7 comes from an eclogite body within the Gneiss complex and contains mm-sized omphacite, garnet and carbonate. Omphacite and garnet frequently have amphibole alteration rims, with some rims also containing chlorite. Rutile has grain sizes of 100–300 µm and often form aggregates of up to 20 grains. The sample has two layers, the first being more mafic, containing clinopyroxene, garnet, quartz, kyanite, amphibole alteration rims, minor white mica and calcite. The second, containing > 50 Vol. % calcite, clinopyroxene, and kyanite. Rutile occurs as 300–700 µm sized grains within the mafic layer.

Granulite Facies Samples

Two samples come from metapelite septa from the Ivrea Zone, Italian Alps. Sample IV16-24 (Val Mastalone) reached peak metamorphic temperatures of > 1000 °C but were largely re-equilibrated at 770 ± 30 °C, while sample IV17-05 (Isola) reached peak metamorphic temperatures of 915 ± 15 °C with Zr-in-Rutile temperatures mostly reset during at 840 ± 30 °C (Ewing et al., 2013). Sample IV16-24 has a mineral assemblage of garnet, quartz, k-feldspar and accessory white mica, while sample IV17-05 contains additionally sillimanite. It is much coarser grained and more dominated

by quartz rather than garnet as in sample IV16-24. Rutile in both samples has grain sizes < 60 µm and occurs homogeneously throughout the matrix and as inclusions in garnet of sample IV17-05.

One sample from the Serre Massif, Calabria, Southern Italy (KINZ) was studied. It belongs to the migmatitic metapelite unit and records peak metamorphic conditions of 0.7 ± 0.1 and 800 ± 50 °C (Schenk, 1984). The sample contains large, up to ~5mm sized garnets in a matrix of cordierite, sillimanite, biotite and plagioclase. Evidence for migmatisation is present throughout the sample. Rutile occurs as fine grained < 100 µm sized grains.

Two samples from the Eastern Ghats, India that relate to PT-conditions of 0.9 ± 0.1 GPa and 1000 ± 50 °C (Mukhopadhyay and Basak, 2009) were studied. Samples AN-1 and AN-2 have a mineral assemblage of quartz, cordierite, orthopyroxene, and spinel. Sample An-1 contains cordierite and quartz grains up to 2 mm size, while sample AN-2 is finer grained. Quartz commonly occurs as elongated lenses. Orthopyroxene is orange-brown in color and forms distinct layers in both samples. Spinel is more abundant in sample AN-2 and occurs as dark green, ~300 µm-sized grains in association with orthopyroxene. Sample AN-2 shows extensive alteration features.

Four HP granulite facies samples from the Bohemian Massif were studied. Three samples from the Sudetes, Poland (WPT-4-1, WPT-4-2B, WPT-10), that had peak metamorphic conditions of 1.9 ± 0.1 GPa and 950 ± 50 °C, (O'Brien et al. 1997; Anczkiewicz et al. 2007) and one sample from the Erzgebirge, Bohemian Massif, Germany (ERZF5b) records peak metamorphic conditions of 1.8 ± 0.4 GPa and 1050 ± 50 °C (O'Brien and Rötzler, 2003). Samples WPT-4-1, WPT-4-2B, and WPT-10 contain mm-sized garnet in a matrix of quartz, k-feldspar, and plagioclase, with the matrix of WPT-10 being significantly finer grained and foliated. Sample WPT-10 additionally contains small kyanite and biotite grains throughout the matrix. All samples show amphibolite-alteration, with WPT-4-1 being the least altered and WPT-10 being extensively altered. Rutile occurs as < 100 µm-sized grains. Sample ERZF5b contains mm-sized, elongated garnet in a matrix of fine grained omphacite and quartz. Omphacite is largely altered to fine grained amphibole and biotite. Rutile occurs as 100–200 µm sized grains throughout the matrix.

Table S1: Sample localities with coordinates, if known.

Locality		Sample	Coordinates
Western Alps	Dora-Maira	DM1c	44°36’26” N 7°20’40” E
		DM2a	44°36'43"N 7°20'52"E
		DM5	44°35'00"N 7°20'29"E
		DM9	44°34'36"N 7°22'06"E
		DM10	44°34'36"N 7°22'06"E
		DM30	44°34'36"N 7°22'26"E
	Lago di Cignana	C31	45°52’44’’ N 7°35’35’’ E
	Pfulwe	PF18-14	46°00’59” N 7°50’53” E
		PF18-25c	46°00’59” N 7°50’53” E
		PF18-26b	46°00’59” N 7°50’53” E
		PF18TB01	46°00’56’’ N 7°50’29’’ E
		PF21-01	46°00’48’’ N 7°50’23’’ E
		PF21-02	46°00’48’’ N 7°50’23’’ E
	Monviso	MVE2	44°41'34"N 7°05'17"E
		MVE4	44°41'34"N 7°05'17"E
		MVE12	44°41'44"N 7°05'16"E
	Monte Mucrone	M-E1	45° 37'48’’ N 7°56’31'' E
		M-E2	45°37'48’’ N 7°56’31'' E
		M-Q1	45°37'48’’ N 7°56’31'' E
		M-Q2	45°37'48’’ N 7°56’31'' E
		M-Q3	45°37'48’’ N 7°56’31'' E
	Ivrea	IV16-04	45°55’50’’ N 8°17’33’’ E
		IV17-05	45°55’50’’ N 8°17’33’’ E (river sample)
Central Alps	Alpe Arami	AA16-11	46°13’46.0” N 8°58’50.1” E
		AA20-1	46°13’46.0” N 8°58’50.1” E
		AA21-01	46°13’46’’ N 8°59’00’’ E
		AA21-02	46°13’46’’ N 8°59’00’’ E

Table S1 continued

Locality		Sample	Coordinates
Central Alps	Cima di Gagnone	CdG19/36	46°19’23’’ N 8°50’45’’ E
	Alpe Capoli	CP16-03B	46°12’52” N 8°43’27” E
		CP16-04	46°12’51’’ N 8°43’32’’ E
		CP18-03A	46°12’53’’ N 8°43’29’’ E
		CP18-03B	46°12’53’’ N 8°43’29’’ E
	Val Cama	CM20-2	46°16'03" N 9°10'19" E
		CM20-6	46°15'59" N 9°11'52" E
	Alpe Senevedo Superiore	AS19-1	loose block
		AS19-2	loose block
		AS19-3	loose block
	Binntal	Rt1	
		Binn	
	Campo Tencia	6059	
	Faulhorn	A7653	
	Mompe Medel	A6758	
	Iragna	A5732	
	Campolungo	19-JH-03	46°28’40’’ N 8°43’38’’ E
Eastern Alps	Koralpe	Kor3	46°43´32’’ N 15°08´44’’ E
	Saualpe	Sau2	46°49´53’’ N 14°37´22’’ E
		Sau3	46°49´53’’ N 14°37´22’’ E
	Ulten Zone	Ulten	
Bohemian Massif	Sudetes	WPT-4-1	
		WPT-4-2B	
		WPT-10	
Brazil	Bahia	GrB	
		SFJ	
West Gondwana Orogen, Mali		S-508	1°40’35’’ N 0°38’49’’ W
		S-520	1°40’35’’ N 0°38’49’’ W
Western Gneiss Region, Norway		WG17-01	
		FJ-RT	62°42’45’’ N 6°25’29’’ E
Serre Massif, Sardinia, Italy		KINZ	
Syros, Cyclades, Greece		SY21-31	37°23’12” N 24°57’02” E
		SY-KM1	
		SY-KM2	

Table S1 continued

Locality	Sample	Coordinates
Eastern Ghats, India	An-1	18° 14'17'' N<br>83° 00'51'' E
	An-2	18° 14'17'' N<br>83° 00'51'' E
Dabie Shan, China	DB1	
	DB2	
	SH02-3	
	DB6	
	DB7	
Alinci, North Macedonia	MP2	
Graves Mountain, Georgia, USA	CYW2320	
Soběslav, Bohemia, Czech Republic	A5088	
